# Supplementary material for: Education mediating the associations between early life factors and frailty: a cross-sectional study of the UK Biobank
Source: BMJ Open. 2023 Mar 2;13(3):e057511. doi: 10.1136/bmjopen-2021-057511 (PMC9990643; doi:10.1136/bmjopen-2021-057511)
Supplement: Supplementary data [file bmjopen-2021-057511supp001.pdf]

Supplementary Material: Pathways linking early life factors and frailty among middle-aged and older adults in England: Findings from UK Biobank

Supplementary Table 1. Variables included in the UK Biobank frailty indices

Item	Variable	Definition	Coding
	Sensory		
1	Glaucoma	Self-report of physician-diagnosed glaucoma	0=no; 1=yes
2	Cataracts	Self-report of physician-diagnosed glaucoma	0=no; 1=yes
3	Hearing difficulty	Self-report experiencing hearing difficulty	0=no; 1=yes/completely deaf
	Cranial		
4	Migraine	Self-report of physician-diagnosed migraine	0=no; 1=yes
5	Dental problems	Self-report of physician-diagnosed dental problems, i.e., ulcers, painful gums, bleeding gums, loose teeth, toothache, dentures	0=none; 1=any
	Mental well-being		
6	Self-rated health	Self-rated health in 4 Likert scale	0=excellent; 0.25=good; 0.5=fair; 1=poor
7	Fatigue	Self-report of frequency of tiredness / lethargy in last two weeks	0=not at all; 0.25=several days; 0.5=more than half; 1=nearly every day
8	Sleep	Self-report experiencing of sleeplessness/ insomnia	0=never/rarely; 0.5=sometimes; 1=usually
9	Depressed feelings	Self-report of frequency having depressed feeling in last two weeks	0=not at all; 0.5=several days; 0.75=more than half; 1=nearly every day
10	Self-described nervous personality	Self-report of having nervous personality	0=no; 1=yes
11	Severe anxiety/ panic attacks	Self-report of physician-diagnosed severe anxiety/panic attacks	0=no; 1=yes
12	Common to feel loneliness	Self-report of feeling lonely commonly	0=no; 1=yes
13	Sense of misery (ever/never)	Self-report of ever having sense of misery	0=no; 1=yes
	Infirmity		
14	Infirmity	Self-report of having long-standing illness or disability	0=no; 1=yes
15	Falls in last year	Self-report of experiencing falls last year	0=no falls; 0.5=one fall; 1=more than one fall
16	Fractures/broken bones in last five years	Self-report of experiencing fractures/broken bones in last five years	0=no; 1=yes
	Cardiometabolic		
17	Diabetes	Self-report of physician-diagnosed diabetes	0=no; 1=yes
18	Myocardial infarction	Self-report of physician-diagnosed myocardial infarction	0=no; 1=yes
19	Angina	Self-report of physician-diagnosed angina	0=no; 1=yes
20	Stroke	Self-report of physician-diagnosed stroke	0=no; 1=yes
21	High blood pressure	Self-report of physician-diagnosed high blood pressure	0=no; 1=yes
22	Hypothyroidism	Self-report of physician-diagnosed hypothyroidism	0=no; 1=yes
23	Deep-vein thrombosis	Self-report of physician-diagnosed deep-vein thrombosis	0=no; 1=yes
24	High cholesterol	Self-report of physician-diagnosed high cholesterol	0=no; 1=yes

	Respiratory		
25	Breathing	Self-report of having wheeze in last year	0=no; 1=yes
26	Pneumonia	Self-report of physician-diagnosed pneumonia	0=no; 1=yes
27	Chronic bronchitis/emphysema	Self-report of physician-diagnosed chronic bronchitis/emphysema	0=no; 1=yes
28	Asthma	Self-report of physician-diagnosed asthma	0=no; 1=yes
	Musculoskeletal		
29	Rheumatoid arthritis	Self-report of physician-diagnosed rheumatoid arthritis	0=no; 1=yes
30	Osteoarthritis	Self-report of physician-diagnosed osteoarthritis	0=no; 1=yes
31	Gout	Self-report of physician-diagnosed gout	0=no; 1=yes
32	Osteoporosis	Self-report of physician-diagnosed osteoporosis	0=no; 1=yes
	Immunological		
33	Hay fever, allergic rhinitis or eczema	Self-report of physician-diagnosed hay fever, allergic rhinitis or eczema	0=no; 1=yes
34	Psoriasis	Self-report of physician-diagnosed psoriasis	0=no; 1=yes
	Cancer		
35	Any cancer diagnosis	Self-report of physician-diagnosed any cancer	0=no; 1=yes
36	Multiple cancers diagnosed (number reported)	Self-report of physician-diagnosed multiple cancer	0=no cancer or single cancer; 1=multiple cancer
	Pain		
37	Chest pain	Self-report of ever experiencing chest pain	0=no; 1=yes
38	Head and/or neck pain	Self-report of ever experiencing head and/or neck pain	0=no; 1=yes
39	Back pain	Self-report of ever experiencing back pain	0=no; 1=yes
40	Stomach/abdominal pain	Self-report of ever experiencing stomach/abdominal pain	0=no; 1=yes
41	Hip pain	Self-report of ever experiencing hip pain	0=no; 1=yes
42	Knee pain	Self-report of ever experiencing knee pain	0=no; 1=yes
43	Whole-body pain	Self-report of ever experiencing whole-body pain	0=no; 1=yes
44	Facial pain	Self-report of ever experiencing facial pain	0=no; 1=yes
45	Sciatica	Self-report of physician-diagnosed sciatica	0=no; 1=yes
	Gastrointestinal		
46	Gastric reflux	Self-report of physician-diagnosed gastric reflux	0=no; 1=yes
47	Hiatus hernia	Self-report of physician-diagnosed hiatus hernia	0=no; 1=yes
48	Gall stones	Self-report of physician-diagnosed gall stones	0=no; 1=yes
49	Diverticulitis	Self-report of physician-diagnosed diverticulitis	0=no; 1=yes

Notes: Deficit points are summed for each individual, and divided by the total number of deficits, to produce a frailty index with a range from 0 to 1.

Supplementary Table 2. Regression models predicting frailty index

	**Non-imputed data[1] (n=190,575)**	**Imputed data[1] (n=502,489)**
Breastfed as a baby	-0.0042 (-0.0048,-0.0035)†	-0.0045 (-0.0051,-0.0038)†
Maternal smoking	0.0118 (0.0111,0.0125)†	0.0122 (0.0116,0.0128)†
Low birthweight	0.0108 (0.0097,0.0118)†	0.0114 (0.0105,0.0122)†
High birthweight	0.0030 (0.0021,0.0039)†	0.0036 (0.0028,0.0044)†
Perinatal diseases	0.0117 (0.0049,0.0185)*	0.0107 (0.0046,0.0167)*
Birth month (cos)	-0.0006 (-0.0011,-0.000)*	-0.0006 (-0.0010,-0.0002)*
Born in the UK	0.0024 (0.0011,0.0037)†	0.0018 (0.0006,0.0030)*
Education	-0.0140 (-0.0147,-0.0134)†	-0.0144 (-0.0150,-0.0139)†
Age (years)	0.0015 (0.0015,0.0015)†	0.0015 (0.0015,0.0016)†
Male	-0.0084 (-0.0090,-0.0078)†	-0.0084 (-0.0090,-0.0079)†
Caucasian ethnicity	-0.0069 (-0.0088,-0.0050)†	-0.0068 (-0.0085,-0.0051)†
Smoking	0.0244 (0.0234,0.0254)†	0.0248 (0.0239,0.0257)†
Alcohol drinking	-0.0260 (-0.0272,-0.0248)†	-0.0268 (-0.0279,-0.0258)†
Physical activity	-0.0065 (-0.0068,-0.0062)†	-0.0069 (-0.0072,-0.0067)†
Intercept	0.0976 (0.0947,0.1005)†	0.0998 (0.0972,0.1024)†

Note: [1] presented are coefficients (95% confidence intervals); *Significant at 0.05; † Significant at 0.0001. Non-imputed analysis was based on 214,104 respondents with complete information on all variables. The maternal smoking variable includes 13.86% missing data, the breastfed as a baby variable includes 23.64% missing data, the birthweight variable includes 44.88% missing data, the education variable includes 2.02% missing data, and the moderate or vigorous physical activity variable includes 2.43% missing data. The imputed analysis included all the respondents (n=502,489).
