# Supplementary material for: Education mediating the associations between early life factors and frailty: a cross-sectional study of the UK Biobank
Source: BMJ Open. 2023 Mar 2;13(3):e057511. doi: 10.1136/bmjopen-2021-057511 (PMC9990643; doi:10.1136/bmjopen-2021-057511)
Supplement: Author's manuscript [file bmjopen-2021-057511.draft_revisions.pdf]

BMJ Open

BMJ Open is committed to open peer review. As part of this commitment we make the peer review history of every article we publish publicly available.

When an article is published we post the peer reviewers' comments and the authors' responses online. We also post the versions of the paper that were used during peer review. These are the versions that the peer review comments apply to.

The versions of the paper that follow are the versions that were submitted during the peer review process. They are not the versions of record or the final published versions. They should not be cited or distributed as the published version of this manuscript.

BMJ Open is an open access journal and the full, final, typeset and author-corrected version of record of the manuscript is available on our site with no access controls, subscription charges or pay-per-view fees (http://bmjopen.bmj.com).

If you have any questions on BMJ Open's open peer review process please email info.bmjopen@bmj.com

BMJ Open

Pathways linking early life factors and frailty among middle-aged and older adults in England: Findings from the UK Biobank

Journal:	*BMJ Open*
Manuscript ID	bmjopen-2021-057511
Article Type:	Original research
Date Submitted by the Author:	19-Sep-2021

Complete List of Authors:	Maharani, Asri; The University of Manchester, Social Work<br>Didikoglu, Altug; The University of Manchester, Division of Neuroscience & Experimental Psychology<br>O'Neill, Terence; University of Manchester, Centre for Epidemiology Versus Arthritis, Division of Musculoskeletal & Dermatological Sciences; Manchester University NHS Foundation Trust, UK & NIHR Biomedical Research Centre<br>Pendleton, Neil; The University of Manchester, Division of Neuroscience & Experimental Psychology<br>Canal, Maria; The University of Manchester, Division of Neuroscience & Experimental Psychology<br>Payton, Antony; The University of Manchester, Division of Informatics, Imaging & Data Sciences

Keywords:	EPIDEMIOLOGY, GERIATRIC MEDICINE, SOCIAL MEDICINE

SCHOLARONE™
Manuscripts

I, the Submitting Author has the right to grant and does grant on behalf of all authors of the Work (as defined in the below author licence), an exclusive licence and/or a non-exclusive licence for contributions from authors who are: i) UK Crown employees; ii) where BMJ has agreed a CC-BY licence shall apply, and/or iii) in accordance with the terms applicable for US Federal Government officers or employees acting as part of their official duties; on a worldwide, perpetual, irrevocable, royalty-free basis to BMJ Publishing Group Ltd ("BMJ") its licensees and where the relevant Journal is co-owned by BMJ to the co-owners of the Journal, to publish the Work in this journal and any other BMJ products and to exploit all rights, as set out in our licence.

The Submitting Author accepts and understands that any supply made under these terms is made by BMJ to the Submitting Author unless you are acting as an employee on behalf of your employer or a postgraduate student of an affiliated institution which is paying any applicable article publishing charge ("APC") for Open Access articles. Where the Submitting Author wishes to make the Work available on an Open Access basis (and intends to pay the relevant APC), the terms of reuse of such Open Access shall be governed by a Creative Commons licence – details of these licences and which Creative Commons licence will apply to this Work are set out in our licence referred to above.

Other than as permitted in any relevant BMJ Author's Self Archiving Policies, I confirm this Work has not been accepted for publication elsewhere, is not being considered for publication elsewhere and does not duplicate material already published. I confirm all authors consent to publication of this Work and authorise the granting of this licence.

For peer review only

Title Page

Title:

Pathways linking early life factors and frailty among middle-aged and older adults in England: Findings from the UK Biobank

Running Title:

Early-life factors and frailty

Keywords:

UK Biobank, Early-life factors, frailty

Authors and author affiliations:

Asri Maharani, MD, PhD[1*], Altug Didikoglu, PhD[2*], Terence W O'Neill, MD, PhD[3], Neil Pendleton, MD, PhD[2], Maria Mercè Canal, PhD[2], Antony Payton, PhD[4]

1) Division of Nursing, Midwifery, and Social Work, The University of Manchester, UK

2) Division of Neuroscience & Experimental Psychology, The University of Manchester, UK

3) Centre for Epidemiology Versus Arthritis, Division of Musculoskeletal & Dermatological Sciences, The University of Manchester, UK & NIHR Biomedical Research Centre, Manchester University NHS Foundation Trust, Manchester.

4) Division of Informatics, Imaging & Data Sciences, The University of Manchester, UK

* These authors contributed equally to this work

Corresponding author's full address and corresponding author's current email:

Name: Asri Maharani

Address: Division of Nursing, Midwifery, and Social Work, The University of Manchester, Jane McFarlane Building, Oxford Road, M13 9PL, Manchester, UK

Email: asri.maharani@manchester.ac.uk

Acknowledgements

For peer review only

Altug Didikoglu was supported by a grant from the Republic of Turkey Ministry of National Education. This research has been conducted using the UK Biobank Resource under Application Number 41877.

Conflicts of interest

All authors declare no conflicts of interest.

Main text word counts: 3296

Number of data elements: 3

For peer review only

Abstract

Objectives Exposures in utero and during infancy may impact the development of diseases later in life. They may be linked with development of frailty though the mechanism is unclear. This study aims to determine the associations between early life risk factors and development of frailty amongst middle-aged and older adults as well as potential pathways via education and socioeconomic factors, for any observed association.

Design A cross-sectional study.

Settings This study used data from UK Biobank, a large population based cohort.

Participants 502,489 individuals aged 37-73 years were included in the analysis.

Primary and secondary outcome measures Early life factors in this study included being breastfed as a baby, maternal smoking, birth weight, the presence of perinatal diseases, birth month, and birth place (in or outside the UK). We developed a frailty index comprising 49 deficits. We used generalised structural equation modelling to examine the associations between early life factors and development of frailty and whether any observed association was mediated via educational attainment and income level.

Results A history of breastfeeding and higher birth weight were associated with a lower frailty index while maternal smoking, the occurrence of perinatal diseases and birth month with a longer day length were associated with a higher frailty index. Both educational level and income mediated the relationship between these early life factors and frailty index.

Conclusions This study highlights that biological and social risk occurring at different stages of life are related to the variations in frailty index in later life and suggests opportunities for prevention across the life course.

Keywords early life factors, frailty, generalised structural equation model, UK Biobank

For peer review only - http://bmjopen.bmj.com/site/about/guidelines.xhtml

Article Summary

Strengths and limitations of this study

- Using a large cohort of British adults in middle and older age, this study was sufficiently powered to identify soociations between early life factors and fralty index.
- The findings provide the first evidence that education and income mediate the association between early life factors and frailty index.
- As the cohort is not nationally representative, the findings cannot be generalised to the general population.
- The questionnaire on early life factors was based on self-report and is therefore subject to recall error.

For peer review only

For peer review only - http://bmjopen.bmj.com/site/about/guidelines.xhtml

Introduction

As the world's population ages, a major goal is the attainment of increased life expectancy accompanied by fewer years spent in poor health and with disability and dependency. The worldwide population of older people (65 years and above) is predicted to double from 0.7 billion (9%) in 2019 to 1.5 billion (16%) in 2050.[1] In addition, there is evidence that the number of disability adjusted life years (DALY) among those aged 60 years and older is increasing (from 434 million in 1990 to 574 million in 2010),[2] which will increase demand for health and care services. As physical disability is an adverse outcome of frailty,[3] more research in geriatrics and gerontology has focused on defining and recognising frailty among older people with the aim of determining preventive and interventional measures.[4]

Frailty can be defined as a state of increased vulnerability resulting from an age-related decline in physiologic and cognitive reserves and function following stressor events.[5] The frailty index approach, developed by Rockwood et al.,[6] measures frailty level as the number of deficits present over the number of deficits considered, including symptoms, diagnosis, disabilities, and functional impairments. Frailty has become more common with the ageing of the population. A systematic review including 240 studies from 62 countries showed that 24% of people aged 50 years and older are frail as calculated using the frailty index approach.[7] Frailty has been found to be associated with adverse health outcomes including loss of mobility, disability, falls, hospitalisation, need for long-term care, and death.[8-10] Understanding the factors that are associated with frailty is thus important for developing interventions to prevent frailty and for providing directions for future public health policies.

A growing body of literature acknowledges that the first two decades of human life are critical in determining adult life trajectories. Body size at birth has been found to be associated with adult chronic diseases,[11,12] grip strength[13] and physical activity.[14] Evidence has suggested that cigarette smoke exposure in utero is linked to the development of chronic diseases later in

For peer review only

For peer review only - http://bmjopen.bmj.com/site/about/guidelines.xhtml

life, including type 2 diabetes, obesity, certain cancers and respiratory disorders.[15] Infants exclusively breastfed have been found to have a lower risk of obesity, type 2 diabetes and high blood pressure in adulthood.[16] Studies in the USA[17] and China[18] have discovered a relationship between birth month and the risk of cardiovascular diseases later in life. In the USA, women born in spring and summer were shown to have higher cardiovascular specific mortality rates than those born in the autumn.[17] In a study using patient medical records from the BioBank of First Affiliated Hospital of Xinxiang Medical University patients born in winter were found to have a greater risk of coronary artery disease than those born in spring.[18] New-borns' perinatal complications are related to accelerated ageing at midlife.[19]

There is some evidence also of a link between early life factors and occurrence of frailty. In a recent study in Finland, greater weight, length and BMI at birth were associated with a lower risk of frailty later in life.[20] However, data from the Netherlands have suggested no significant association between prenatal undernutrition and frailty among older adults.[14] The present study thus aims to determine the associations between early life factors, including a history of being breastfed, maternal smoking, birth weight, the presence of perinatal diseases, birth in or outside of the UK or outside the UK and birth month, and frailty in UK adults.

Furthermore, this study contributes to the literature investigating the determinants of health in later life by exploring the pathways of early life factors that have a lasting impact on health in middle and old age. The pathway hypothesis posits that early life conditions are important not only because they are directly associated with late life but also because they shape later life experiences,[21,22] including restricted educational attainment and life chances. The most frequently hypothesised pathway between circumstances in early stages of life and adult health is adult socioeconomic status. Pakpahan et al. showed that education and income mediate the link between childhood health and socioeconomic conditions and self-rated health among older Europeans.[21] Maternal smoking during pregnancy was found to be correlated

For peer review only - http://bmjopen.bmj.com/site/about/guidelines.xhtml

with the children's cognitive function,[23] while a longitudinal study in the US documented the relationship between low birth weight and lower educational attainment.[24] Education and income are among the predictors of frailty.[25,26] Because interventions that target common pathways have the potential to reduce frailty, the identification of the pathways of early life factors leading to frailty later in life has substantial public health relevance for the translation of life course epidemiology into practice. The present study considers whether any observed association between early life factors and frailty could be attributed to differences in education attainment and adult socioeconomic status (Figure 1).

Methods

Source and Sample

Data were drawn from the UK Biobank, a prospective cohort study of the genetic, environmental and lifestyle causes of diseases among adults in the UK.[27] The study involved the collection of extensive questionnaire data and biological samples from, and the performance of, physical examinations of more than 500,000 respondents enrolled at 22 assessment sites in England, Scotland, and Wales between 2006 and 2010. Subjects who took part provided written informed consent for data collection, analysis and linkage; they also completed a touchscreen questionnaire, a nurse-led interview, and had their physical measurements taken. The UK Biobank invited adults who were registered with a general practitioner and who lived within reasonable traveling distance of the assessment centre. The current study includes 502,489 individuals aged 37-73 years who had study-specific available data and were not withdrawn from the study. This study was conducted as part of UK Biobank Project Number 41877 and is covered by the generic ethics approval for UK Biobank studies from the NHS National Research Ethics Service (16/NW/0274).

Measures

Early life factors

For peer review only

Information by questionnaire was obtained on: maternal smoking in the pre- and post-natal period, history of being breastfed as a baby, birth month, birthweight, the presence of perinatal diseases, and place of birth. We defined maternal smoking based on the question 'Did your mother smoke regularly around the time when you were born?' (Data-Field 1787). Respondents were categorised as having been breastfed as babies if they answered 'yes' to the question: 'Were you breastfed when you were a baby?' (Data-Field 1677). We retrieved information on birth month from the birth date (Data-Field 52) and treated it as the cosine of the values, representing the rhythmic seasonal length of day and night. We considered this might represent daylight time better than treating it as a categorical variable. This is an approach which we have used in a previous study.[28] Birth months of participants born in the UK and other countries in the southern hemisphere were converted to their antiphase. Information on birthweight was gathered by means of self-reported birthweight in kilograms (Data-Field 20022). We used the same measure in our prior study.[28] The presence of perinatal diseases ('ICD10 Chapter XVI: Certain conditions originating in the perinatal period') was coded as one based on self-reported medical history (Category 2416). We categorised the place birth of the respondents as born in the UK or outside the UK (Data-Field 1647). Answers of 'Do not know' or 'Prefer not to answer' were accepted as missing for all questions.

Education

The education variable represents the highest educational level completed by the respondents. Qualifications were categorised as high school or less (reference) and college or university degree (Data-Field 6138).

Income

Income (at the time of the assessment) was determined according to average total household income before tax (Data-Field 738). Income was classified into five ordinal groups: less than

For peer review only - http://bmjopen.bmj.com/site/about/guidelines.xhtml

£18,000; £18,000 to £30,999; £31,000 to £51,999; £52,000 to £100,000; and greater than £100,000.

Frailty index

Following William et al.,[29] we derived the frailty index using 49 functional, psychological, and social deficits within the range of data variables in the UK Biobank (see Supplementary Table 1). We coded the binary variables as 0 or 1, and for ordinal and continuous variables, coding was based on distribution. The total number of deficits was summed and divided by total possible deficits to create a frailty index between 0 and 1, where higher scores indicated greater frailty.

Covariates

We included demographic and health behaviour as covariates. Demographic information included age (in years; Data-Field 21003), gender (with male as the reference; Data-Field 31), and ethnicity (other than Caucasian as the reference or Caucasian; Data-Field 21000). Health behaviours included physical activity, alcohol intake and smoking status. Physical activity was measured as the number of days per week respondents engaged in at least 10 minutes of moderate or vigorous physical activity (Data-Field 884, Data-Field 904). Respondents were classified as non-current smokers (reference) or current smokers (Data-Field 20116). Alcohol intake status was classified as non-current (reference) or current alcohol drinking (Data-Field 20117).

Statistical Analyses

Descriptive statistics were used to summarise subject characteristics including means and standard deviation for continuous variables and frequencies and percentages for categorical variables. We looked at the associations between frailty index and both early life factors and other covariates using unpaired t-tests (dichotomous variables), ANOVA (categorical variables), and Pearson's correlation (continuous variables).

For peer review only - http://bmjopen.bmj.com/site/about/guidelines.xhtml

For peer review only

The structural equation model (SEM) has been widely used to investigate complex relationships between variables in epidemiological studies.[30] SEM can be used to resolve the endogeneity problem between variables and to explore direct, indirect, and total effects between exogenous and endogenous variables. It can jointly test a variety of hypotheses that involve different types of complicated cause-effect relationships. However, all responses are assumed to be continuous, even when a variable is binary or categorical. In our analysis we include binary (education) and ordinal outcome variables (income). To address this, we used a generalised structural equation model (GSEM) to identify the link between early life factors and frailty index and the mediating effect of education and income on that relationship. A GSEM combines generalised linear model (GLM) estimation and SEM modelling estimation; it can accommodate binary, ordinal, counted and categorical data.[31] Using maximum likelihood estimators, GLM estimators are based on a density function, allowing the direct use of all types of data.[32] The analyses were performed using the 'gsem' command (STATA Version 17).

We examined two models. In Model 1, we considered early life factors and frailty index. We then added education and income as mediators of the relationship in Model 2. All models were controlled for age, gender and health behaviours. Modification indices and model fit estimates are not facilitated in the '*gsem*' command in STATA. The binary endogenous variable and paths are thus interpreted by their level of significance.

We performed two sensitivity analyses. We first performed regression models including early life factors, education, income, and covariates. We further handled missing data using multivariate imputation by chained equations (MICE)[33] (using Stata's mi program).[34] Twenty imputations were used. In the second supplementary analysis, we performed the same GSEM models using participants aged 60 years and over.

Patient and public involvement

For peer review only - http://bmjopen.bmj.com/site/about/guidelines.xhtml

Patients and/or the public were not involved in this study.

Results

Subjects

The study sample consisted of 502,489 respondents with an average age of 56.53 years (standard deviation [SD]=8.10 years) (Table 1). Just under half (45%) of the respondents were male, and most were Caucasian (94.59%). Around one-third of the respondents had graduated from college or university. The proportion of respondents whose mothers smoked regularly around the time of their birth was 29%. More than 72% of respondents were breastfed as babies, and 0.18% had perinatal diseases. The average birth weight was 3.32 kg (SD=0.67 kg). 91% of the respondents were born in the UK. Just over two-thirds of subjects reported engaging in at least 10 minutes of moderate or vigorous physical activity at least three days per week; 91% consumed alcohol and 10% were current smokers.

Early life factors, covariates and frailty index

In bivariate analyses, compared to those whose mothers did not smoke around birth, maternal pre and post-natal smoking was associated with a significantly higher frailty index (0.146 vs 0.133) as was the presence of perinatal diseases (0.149 vs 0.138) and being born in the UK (0.138 vs 0.137). A history of breast feeding was associated with a lower frailty index (0.134 vs 0.137). Higher birth weight (r=-0.05) and shorter daylight hours at birth (r=-0.01) were both associated with lower frailty indices. As expected, the frailty index was higher among women than among men and in those with lower educational attainment and lower income. The frailty index was also higher in smokers, non-drinkers and those who engaged in less physical activity.

In multivariate analysis and adjusting for age, gender and health behaviours, being breastfed as a baby, higher birth weight, and a birth month with fewer hours of daylight were associated

For peer review only - http://bmjopen.bmj.com/site/about/guidelines.xhtml

with a significantly lower frailty index, while maternal smoking, perinatal diseases and born in the UK had positive and significant associations with frailty index (see Figure 2A).

Mediation analysis

In the second model, education and income mediated the association between early life factors and frailty index among middle-aged and older adults, supporting the pathway hypothesis (Figure 2B). The direct effects of early life factors were diminished in comparison to the previous model. Still, the associations between being breastfed as a baby (coef.=-0.0034, z=-9.49), maternal smoking (coef.=0.0116, z=33.02), birthweight (coef.=-0.0023, z=-9.32) and frailty index remained significant after introducing adulthood characteristics. The presence of perinatal diseases (coef.=0.0132, z=3.69) and birth months with long daylight hours (coef.=-0.0007, z=-3.07) had a relatively small though significant effect on frailty (see Supplementary Table 2).

Education and income mediated the links between early life factors and frailty index. Participants born in the UK had a lower probability of completing higher education (coef.=-0.7993, z=-46.07). Having been breastfed as a baby (coef.=0.1686, z=16.86) and higher birth weight (coef.=0.0779, z=11.11) were associated with higher educational attainment, while maternal smoking was associated with lower educational attainment (coef.=-0.3228, z=-31.43). Higher education was the determinant of income (coef.=1.2703, z=143.32). However, maternal smoking showed no direct relationship with income. Higher birthweight was directly associated with higher income (coef.=0.1312, z=20.46), while having been breastfed as a baby was interestingly associated with lower income (coef.=-0.1086, z=-12.16). Higher average income was significantly associated with a lower frailty index (coef.=-0.0127, z=-84.98). Higher education was also associated with a lower frailty index (coef.=-0.0051, z=-14.64). Amongst covariates with greater effect sizes, older age (coef.=0.0010, z=45.11), lower

For peer review only

For peer review only - http://bmjopen.bmj.com/site/about/guidelines.xhtml

activity levels (coef.=-0.0067, z=-42.98) and smoking (coef.=0.0192, z=35.63) were associated with a higher frailty index.

In sensitivity analyses the effects of early life factors and covariates on the frailty index appeared similar in terms of both magnitude and direction when using both non-imputed and imputed data (see Supplementary Table 3). In a further analysis we included participants who were 60 years and older and found that the results were broadly similar (see Supplementary Table 4).

Discussions

Using data from UK Biobank we found that a history of breastfeeding and higher birth weight were associated with a lower frailty index, while maternal smoking, perinatal diseases and birth month with longer day length were associated with a higher frailty index. This study provides the first evidence that educational attainment level and income may mediate the association between early life factors and frailty index.

Our findings are in keeping with findings from a previous study linking birth weight and frailty index.[20] Our study suggests also an association between other life factors, including maternal smoking, perinatal diseases, and birth month, and frailty index in middle-aged and older adults. Early life factors have previously been linked with higher chronic disease risk later in life.[35] Our findings highlight the importance of early life factors in determining frailty in middle age and older men.

The addition of education and income as mediating variables in this study did not annul the direct effect between early life factors and frailty index. The effects of early life factors on the frailty index persist notwithstanding demographic and health behaviours. In line with our findings, Bleker and colleagues found that prenatal undernutrition is associated with poorer health in old age, including slower gait speed and lower physical functioning and the findings

For peer review only - http://bmjopen.bmj.com/site/about/guidelines.xhtml

remained significant after inclusion of an extensive set of control variables including adult socioeconomic status.[14]

In our analysis we observed that education levels mediate the link between early life factors and the frailty index. Early life factors have a significant relationship with educational attainment, and higher education attainment is linked to lower frailty index. This result is broadly in keeping with a prior study in Sweden which found that the associations between childhood conditions and various old age health indicators (musculoskeletal disorders, cardiovascular disease, self-rated health and impaired mobility) are mediated by education.[36] We found that education has also a partly direct and partly indirect association with frailty index through income. This result is consistent with prior research on the biological and psychological pathways that link childhood health and socioeconomic conditions to self-reported health status among older adults in 15 European countries.[21] Early life health is marked by developmental plasticity; life-course trajectories of socioeconomic attainment could be altered by physical and social conditions[37] and set cascading physiological processes in motion, impacting health decades later.[38]

Our findings have potential implications for policies aiming at preventing frailty among older adults. Subsequent circumstances mediate the impact of early life factors on frailty later in life, and our study suggests that interventions such as improving education in midlife may mitigate early life disadvantages.

To our knowledge, this is the first study to examine mechanisms of the relationships between early life factors, i.e. maternal smoking, having been breastfed as a baby, low birth weight, perinatal diseases, and birth month, and the occurrence of frailty. Our findings are based on a large and well characterised cohort. There are, however, a number of limitations to be consider in interpreting the results. In this study, information concerning early life factors was based on self-report and is therefore subject to recall error. The likely effect of such error would be

For peer review only - http://bmjopen.bmj.com/site/about/guidelines.xhtml

to underestimate the relationship between these factors and the frailty index. Data on income level was based on current income and may not necessarily represent income over the lifecourse; furthermore, it is possible that the presence of ill health reflected in a higher frailty index may have *resulted* in reduced income rather than being a cause of it. Another limitation is that we have limited access to the health conditions of the parents. A broad range of conditions which are comprised in the frailty index bear a hereditary risk, thus taking in account for the health conditions of the parents is important in assessing the independent associations with frailty. Future studies may include the health conditions of the parents as the covariates. Finally, the data were based on a sample of predominantly Caucasian men and women and should be extrapolated beyond this group with caution.[39]

In conclusion, this study indicates an association between early life factors and frailty later in life. Early life conditions are important as the start of a mediated, incremental process during the life course. A comprehensive understanding of the determinants of frailty among middle-aged and older adults requires attention to exposures throughout the entire life course, with a special focus on the in utero and infancy stages and the chains of associated socioeconomic conditions that that connect over the life course. Applying a life course perspective on health in adulthood and old age should have implications for public health interventions, social policy, and further research. Early life is not the only period for any potential successful intervention; as our findings show, early life disadvantages may be offset by education and material wealth. Interventions throughout the life course, and especially during early life, could substantially reduce the health burden later in life.

For peer review only - http://bmjopen.bmj.com/site/about/guidelines.xhtml

Contributorship statement

A.D and A.M performed the data analysis. A.D and A.M drafted the manuscript. N.P, T.W.O, M.M.C., A.P. and were involved in planning and supervised the work. All authors discussed the results and commented on the manuscript.

Competing interest

All authors declare no conflicts of interest.

Funding

Altug Didikoglu was supported by a grant from the Republic of Turkey Ministry of National Education. This research has been conducted using the UK Biobank Resource under Application Number 41877.

Data sharing statement

Data are available in a public, open access repository. Other researchers can apply for UK Biobank data to answer specific research questions.

For peer review only

For peer review only - http://bmjopen.bmj.com/site/about/guidelines.xhtml

References

1. United Nations. *World Population Prospects 2019: Highlights*. New York: Department of Economic and Social Affairs; 2019. Contract No.: (ST/ESA/SER.A/423).

2. Prince MJ, Wu F, Guo Y, et al. The burden of disease in older people and implications for health policy and practice. *Lancet*. 2015;385:549-562.

3. Sternberg SA, Schwartz AW, Karunananthan S, Bergman H, Clarfield MA. The identification of frailty: a systematic literature review. *J Am Geriatr Soc*. 2011;59:2129-2138.

4. Daniels R, van Rossum E, de Witte L, Kempen GI, van den Heuvel W. Interventions to prevent disability in frail community-dwelling elderly: a systematic review. *BMC Health Serv Res*. 2008;8:278.

5. Hogan DB. Models, definitions, and criteria for frailty. *Conn's Handbook of Models for Human Aging*. 2018:35-44.

6. Rockwood K, Mitnitski A. Frailty defined by deficit accumulation and geriatric medicine defined by frailty. *Clin Geriatr Med*. 2011;27:17-26.

7. O'Caoimh R, Sezgin D, O'Donovan MR, et al. Prevalence of frailty in 62 countries across the world: a systematic review and meta-analysis of population-level studies. *Age and Ageing*. 2021;50:96-104.

8. Hanlon P, Nicholl BI, Jani BD, Lee D, McQueenie R, Mair FS. Frailty and pre-frailty in middle-aged and older adults and its association with multimorbidity and mortality: a prospective analysis of 493 737 UK Biobank participants. *Lancet Public Health*. 2018;3:e323-e332.

9. Cegri F, Orfila F, Abellana RM, Pastor-Valero M. The Impact of Frailty on Admission to Home Care Services And Nursing Homes: Eight-Year Follow-Up

For peer review only

For peer review only - http://bmjopen.bmj.com/site/about/guidelines.xhtml

Of A Community-Dwelling, Older Adult, Spanish Cohort. *BMC Geriatrics*. 2020;20:1-13.

10. Ensrud KE, Ewing SK, Taylor BC, et al. Frailty and risk of falls, fracture, and mortality in older women: the study of osteoporotic fractures. *J Gerontol A Biol Sci Med Sci*. 2007;62:744-51.

11. Barker DJ. Fetal origins of coronary heart disease. *BMJ*. 1995;311:171-4.

12. Eriksson JG. Developmental origins of health and disease–from a small body size at birth to epigenetics. *Ann Med.* 2016;48:456-467.

13. Kuh D, Hardy R, Butterworth S, et al. Developmental origins of midlife grip strength: findings from a birth cohort study. *J Gerontol A Biol Sci Med Sci*. 2006;61:702-6.

14. Bleker LS, de Rooij SR, Painter RC, van der Velde N, Roseboom TJ. Prenatal undernutrition and physical function and frailty at the age of 68 years: the Dutch Famine Birth Cohort Study. J *Gerontol A Biol Sci Med Sci*. 2016;71:1306-14.

15. Doherty S, Grabowski J, Hoffman C, Ng S, Zelikoff J. Early life insult from cigarette smoke may be predictive of chronic diseases later in life. *Biomarkers.* 2009;14 Suppl 1:97-101.

16. Horta BL, Loret de Mola C, Victora CG. Long-term consequences of breastfeeding on cholesterol, obesity, systolic blood pressure and type 2 diabetes: a systematic review and meta-analysis. *Acta Paediatr*. 2015;104:30-7.

17. Zhang Y, Devore EE, Strohmaier S, Grodstein F, Schernhammer ES. Birth month, birth season, and overall and cardiovascular disease mortality in US women: prospective cohort study. BMJ. 2019;367.

For peer review only

For peer review only - http://bmjopen.bmj.com/site/about/guidelines.xhtml

18. Zhang B-B, Zhao G-A, Yang M, et al. Birth month associates with risk of coronary artery disease and its complications: A propensity score matched analysis. *Med Clin (Barc)*. 2019;153:454-459.

19. Shalev I, Caspi A, Ambler A, et al. Perinatal complications and aging indicators by midlife. *Pediatrics*. 2014;134:e1315-e23.

20. Haapanen M, Perälä M, Salonen M, et al. Early life determinants of frailty in old age: the Helsinki Birth Cohort Study. *Age and Ageing*. 2018;47:569-75.

21. Pakpahan E, Hoffmann R, Kröger H. The long arm of childhood circumstances on health in old age: Evidence from SHARELIFE. Advances in Life Course Research. 2017;31:1-10.

22. Power C, Hertzman C. Social and biological pathways linking early life and adult disease. *Br Med Bull*. 1997;53(1):210-21.

23. Julvez J, Ribas-Fitó N, Torrent M, Forns M, Garcia-Esteban R, Sunyer J. Maternal smoking habits and cognitive development of children at age 4 years in a population-based birth cohort. *Int J Epidemiol*. 2007;36(4):825-32.

24. Conley D, Bennett NG. Is biology destiny? Birth weight and life chances. American Sociological Review. 2000:458-67.

25. Lang IA, Hubbard RE, Andrew MK, Llewellyn DJ, Melzer D, Rockwood K. Neighborhood deprivation, individual socioeconomic status, and frailty in older adults. *J Am Geriatr Soc*. 2009;57:1776-80.

26. Etman A, Burdorf A, Van der Cammen TJ, Mackenbach JP, Van Lenthe FJ. Socio-demographic determinants of worsening in frailty among community-dwelling older people in 11 European countries. *J Epidemiol Community Health*. 2012;66:1116-1121.

For peer review only - http://bmjopen.bmj.com/site/about/guidelines.xhtml

27. Sudlow C, Gallacher J, Allen N, et al. UK biobank: an open access resource for identifying the causes of a wide range of complex diseases of middle and old age. PLoS Medicine. 2015;12:e1001779.

28. Didikoglu A, Maharani A, Pendleton N, Canal MM, Payton A. Early life factors and COVID-19 infection in England: A prospective analysis of UK Biobank participants. *Early Human Development*. 2021;155:105326.

29. Williams DM, Jylhävä J, Pedersen NL, Hägg S. A frailty index for UK Biobank participants. *J Gerontol A Biol Sci Med Sci*. 2019;74:582-587.

30. Amorim LDAF, Fiaccone RL, Santos CAS, et al. Structural equation modeling in epidemiology. *Cad Saude Publica*. 2010;26:2251-2262.

31. Zhang L, Zhang J. Perception of small tourism enterprises in Lao PDR regarding social sustainability under the influence of social network. Tourism Management. 2018;69:109-120.

32. McCullagh P. Generalized linear models: Routledge; 2019.

33. Azur MJ, Stuart EA, Frangakis C, Leaf PJ. Multiple imputation by chained equations: what is it and how does it work? *Int J Methods Psychiatr Res*. 2011;20:40–49.

34. StataCorp. Stata Multiple-Imputation Reference Manual Release 15. College Station, TX2017.

35. Michels KB. Early life predictors of chronic disease. *J Womens Health (Larchmt)*. 2003;12:157-61.

36. Berndt H, Fors S. Childhood living conditions, education and health among the oldest old in Sweden. *Ageing and Society*. 2016;36:631.

37. Haas SA, Glymour MM, Berkman LF. Childhood health and labor market inequality over the life course. *J Health Soc Behav*. 2011;52:298-313.

For peer review only

For peer review only - http://bmjopen.bmj.com/site/about/guidelines.xhtml

38. Montez JK, Hayward MD. Early life conditions and later life mortality. International Handbook of Adult Mortality: Springer; 2011. p. 187-206.

39. Fry A, Littlejohns TJ, Sudlow C, et al. Comparison of sociodemographic and health-related characteristics of UK Biobank participants with those of the general population. *Am J Epidemiol*. 2017;186:1026-1034.

For peer review only

For peer review only - http://bmjopen.bmj.com/site/about/guidelines.xhtml

List of the figures

Figure 1 The pathways of early life factors and impact on frailty among adults

Figure 2 Generalised structural equation models to identify (A) the association between early life factors and frailty index and (B) education and income as mediators of the relationship between early life factors and frailty index. Note: *Significant at 0.05; † Significant at 0.0001

For peer review only

For peer review only - http://bmjopen.bmj.com/site/about/guidelines.xhtml

Table 1. Subject characteristics (n=502,489)

Variable	Percentage or mean (SD)*	Mean (SD) of frailty index**	Bivariate association with frailty index***
Frailty index, mean (SD)	0.14(0.08)		
Early-life factors			
Maternal smoking around birth, %			p<0.0001
No	70.75%	0.133(0.073)	
Yes	29.25%	0.146(0.078)	
Breastfed as a baby, %			p<0.0001
No	27.65%	0.137(0.076)	
Yes	72.35%	0.134(0.074)	
Birthweight (kg), mean (SD)	3.32(0.67)		R=-0.05, p<0.0001
Birth month, %			p=0.0002
January	8.44%	0.138(0.076)	
February	7.96%	0.137(0.075)	
March	8.98%	0.138(0.075)	
April	8.59%	0.139(0.076)	
May	8.98%	0.138(0.076)	
June	8.45%	0.139(0.076)	
July	8.48%	0.139(0.076)	
August	8.24%	0.138(0.076)	
September	8.14%	0.138(0.075)	
October	8.06%	0.137(0.076)	
November	7.63%	0.137(0.075)	
December	8.03%	0.138(0.076)	
Perinatal diseases, %			p<0.0001
No	99.82%	0.138(0.075)	
Yes	0.18%	0.149(0.084)	
Born in the UK, %			p=0.0381
No	8.96%	0.137(0.076)	
Yes	91.04%	0.138(0.075)	
Sociodemographics			

For peer review only

Age (years), mean (SD)	56.53(8.10)		R=0.16, p<0.0001
Gender, %			p<0.0001
Female	54.40%	0.141(0.075)	
Male	45.60%	0.134(0.075)	
Ethnicity, %			p<0.0001
Other	5.41%	0.140(0.078)	
Caucasian	94.59%	0.137(0.075)	
Education, %			p<0.0001
Less than college	67.27%	0.145(0.077)	
College or university degree	32.73%	0.122 (0.068)	
Average total household income before tax, %			p<0.0001
Less than £18,000	22.85%	0.171(0.083)	
£18,000 to £30,999	25.43%	0.140(0.072)	
£31,000 to £51,999	26.04%	0.125(0.067)	
£52,000 to £100,000	20.28%	0.113(0.062)	
Greater than £100,000	5.39%	0.102(0.059)	
Health behaviours			
Moderate or vigorous physical activity, %			p<0.0001
None	10.75%	0.160(0.084)	
1 day	7.11%	0.133(0.072)	
2 days	13.40%	0.132(0.072)	
3 days or more	68.75%	0.134(0.073)	
Current alcohol consumption, %			p<0.0001
No	8.08%	0.166(0.087)	
Yes	91.92%	0.135(0.073)	
Current smoking, %			p<0.0001
No	89.39%	0.135(0.074)	
Yes	10.61%	0.159(0.083)	

Note: * Presented are means (standard deviation) for continuous variables and percentages for categorical variables. The maternal smoking variable includes 13.86% missing data, the breastfed as a baby variable includes 23.64% missing data, the birthweight variable includes

For peer review only - http://bmjopen.bmj.com/site/about/guidelines.xhtml

44.88% missing data, the education variable includes 2.02% missing data, the average total household income before tax variable includes 15.36% missing data, and the moderate or vigorous physical activity variable includes 2.43% missing data. ** Presented are the means (standard deviation) of the frailty index per group. *** Bivariate analyses are unpaired t-tests for binary variables, ANOVA for ordinal variables, and Pearson's correlation for continuous variables.

For peer review only

For peer review only - http://bmjopen.bmj.com/site/about/guidelines.xhtml

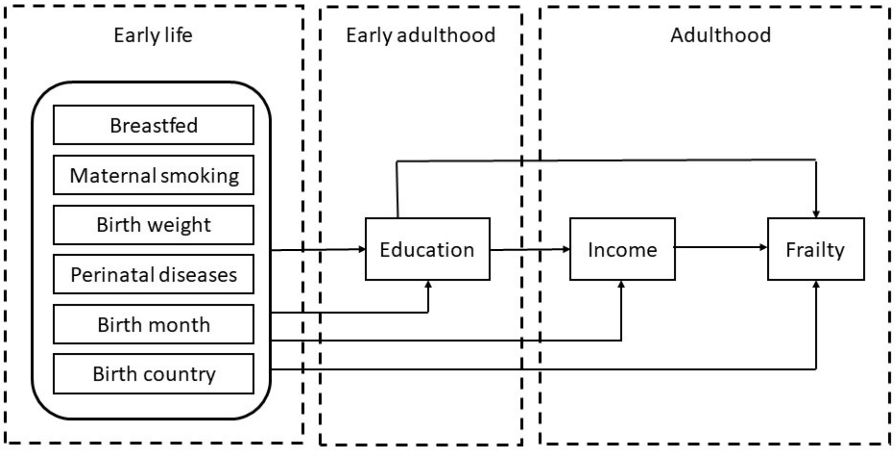

Figure 1 The pathways of early life factors and impact on frailty among adults

275x190mm (96 x 96 DPI)

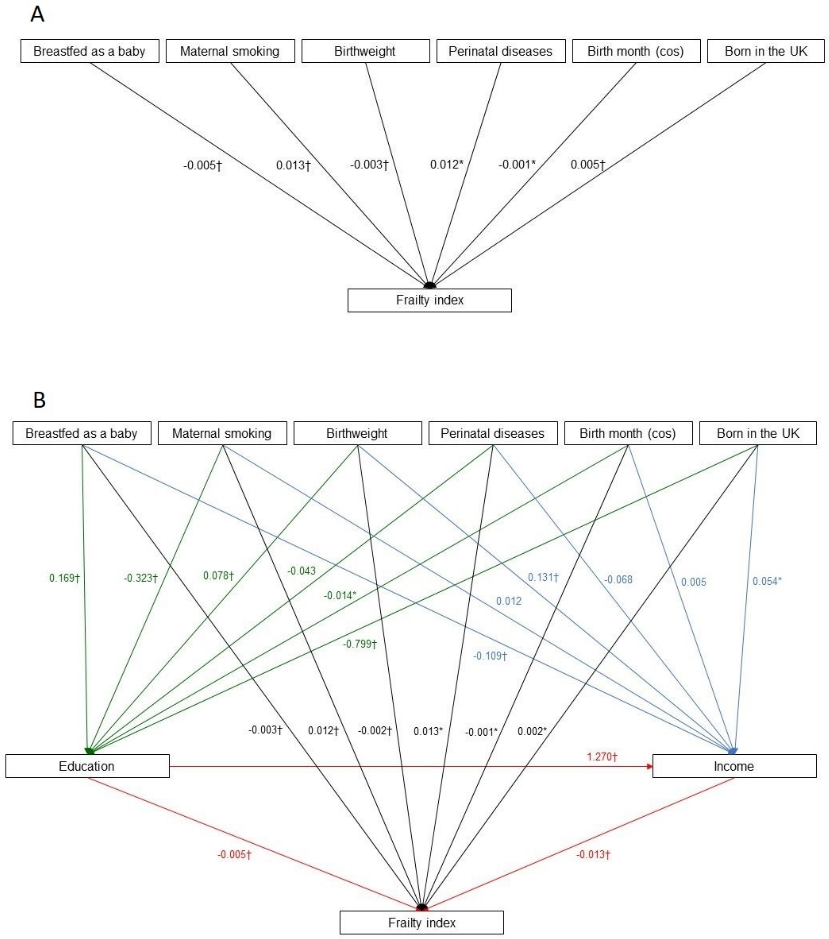

Figure 2 Generalised structural equation models to identify (A) the association between early life factors and frailty index and (B) education and income as mediators of the relationship between early life factors and frailty index. Note: *Significant at 0.05; † Significant at 0.0001

279x399mm (96 x 96 DPI)

Supplementary Material: Pathways linking early life factors and frailty among middle-aged and older adults in England: Findings from UK Biobank

Supplementary Table 1. Variables included in the UK Biobank frailty indices

Item	Variable	Definition	Coding
	Sensory		
1	Glaucoma	Self-report of physician-diagnosed glaucoma	0=no; 1=yes
2	Cataracts	Self-report of physician-diagnosed glaucoma	0=no; 1=yes
3	Hearing difficulty	Self-report experiencing hearing difficulty	0=no; 1=yes/completely deaf
	Cranial		
4	Migraine	Self-report of physician-diagnosed migraine	0=no; 1=yes
5	Dental problems	Self-report of physician-diagnosed dental problems, i.e., ulcers, painful gums, bleeding gums, loose teeth, toothache, dentures	0=none; 1=any
	Mental well-being		
6	Self-rated health	Self-rated health in 4 Likert scale	0=excellent; 0.25=good; 0.5=fair; 1=poor
7	Fatigue	Self-report of frequency of tiredness / lethargy in last two weeks	0=not at all; 0.25=several days; 0.5=more than half; 1=nearly every day
8	Sleep	Self-report experiencing of sleeplessness/ insomnia	0=never/rarely; 0.5=sometimes; 1=usually
9	Depressed feelings	Self-report of frequency having depressed feeling in last two weeks	0=not at all; 0.5=several days; 0.75=more than half; 1=nearly every day
10	Self-described nervous personality	Self-report of having nervous personality	0=no; 1=yes
11	Severe anxiety/ panic attacks	Self-report of physician-diagnosed severe anxiety/panic attacks	0=no; 1=yes
12	Common to feel loneliness	Self-report of feeling lonely commonly	0=no; 1=yes
13	Sense of misery (ever/never)	Self-report of ever having sense of misery	0=no; 1=yes
	Infirmity		
14	Infirmity	Self-report of having long-standing illness or disability	0=no; 1=yes
15	Falls in last year	Self-report of experiencing falls last year	0=no falls; 0.5=one fall; 1=more than one fall
16	Fractures/broken bones in last five years	Self-report of experiencing fractures/broken bones in last five years	0=no; 1=yes
	Cardiometabolic		
17	Diabetes	Self-report of physician-diagnosed diabetes	0=no; 1=yes
18	Myocardial infarction	Self-report of physician-diagnosed myocardial infarction	0=no; 1=yes
19	Angina	Self-report of physician-diagnosed angina	0=no; 1=yes
20	Stroke	Self-report of physician-diagnosed stroke	0=no; 1=yes
21	High blood pressure	Self-report of physician-diagnosed high blood pressure	0=no; 1=yes
22	Hypothyroidism	Self-report of physician-diagnosed hypothyroidism	0=no; 1=yes
23	Deep-vein thrombosis	Self-report of physician-diagnosed deep-vein thrombosis	0=no; 1=yes
24	High cholesterol	Self-report of physician-diagnosed high cholesterol	0=no; 1=yes

For peer review only

For peer review only - http://bmjopen.bmj.com/site/about/guidelines.xhtml

	Respiratory		
25	Breathing	Self-report of having wheeze in last year	0=no; 1=yes
26	Pneumonia	Self-report of physician-diagnosed pneumonia	0=no; 1=yes
27	Chronic bronchitis/emphysema	Self-report of physician-diagnosed chronic bronchitis/emphysema	0=no; 1=yes
28	Asthma	Self-report of physician-diagnosed asthma	0=no; 1=yes
	Musculoskeletal		
29	Rheumatoid arthritis	Self-report of physician-diagnosed rheumatoid arthritis	0=no; 1=yes
30	Osteoarthritis	Self-report of physician-diagnosed osteoarthritis	0=no; 1=yes
31	Gout	Self-report of physician-diagnosed gout	0=no; 1=yes
32	Osteoporosis	Self-report of physician-diagnosed osteoporosis	0=no; 1=yes
	Immunological		
33	Hay fever, allergic rhinitis or eczema	Self-report of physician-diagnosed hay fever, allergic rhinitis or eczema	0=no; 1=yes
34	Psoriasis	Self-report of physician-diagnosed psoriasis	0=no; 1=yes
	Cancer		
35	Any cancer diagnosis	Self-report of physician-diagnosed any cancer	0=no; 1=yes
36	Multiple cancers diagnosed (number reported)	Self-report of physician-diagnosed multiple cancer	0=no cancer or single cancer; 1=multiple cancer
	Pain		
37	Chest pain	Self-report of ever experiencing chest pain	0=no; 1=yes
38	Head and/or neck pain	Self-report of ever experiencing head and/or neck pain	0=no; 1=yes
39	Back pain	Self-report of ever experiencing back pain	0=no; 1=yes
40	Stomach/abdominal pain	Self-report of ever experiencing stomach/abdominal pain	0=no; 1=yes
41	Hip pain	Self-report of ever experiencing hip pain	0=no; 1=yes
42	Knee pain	Self-report of ever experiencing knee pain	0=no; 1=yes
43	Whole-body pain	Self-report of ever experiencing whole-body pain	0=no; 1=yes
44	Facial pain	Self-report of ever experiencing facial pain	0=no; 1=yes
45	Sciatica	Self-report of physician-diagnosed sciatica	0=no; 1=yes
	Gastrointestinal		
46	Gastric reflux	Self-report of physician-diagnosed gastric reflux	0=no; 1=yes
47	Hiatus hernia	Self-report of physician-diagnosed hiatus hernia	0=no; 1=yes
48	Gall stones	Self-report of physician-diagnosed gall stones	0=no; 1=yes
49	Diverticulitis	Self-report of physician-diagnosed diverticulitis	0=no; 1=yes

Notes: Deficit points are summed for each individual, and divided by the total number of deficits, to produce a frailty index with a range from 0 to 1.

For peer review only

For peer review only - http://bmjopen.bmj.com/site/about/guidelines.xhtml

Supplementary Table 2. Generalized structural equation models of frailty

		Model 1 Coef.(95%CI);z	*Model 2* Coef.(95%CI);z
Frailty		N=216,947	N=190,575
	Breastfed as a baby	-0.0052(-0.0059,-0.0046)†;z=-15.3833	-0.0034(-0.0041,-0.0027)†;z=-9.4877
	Maternal smoking	0.0129(0.0122,0.0135)†;z=37.8771	0.0116(0.0109,0.0123)†;z=33.0189
	Birthweight (kg)	-0.0031(-0.0036,-0.0026)†;z=-12.9125	-0.0023(-0.0028,-0.0018)†;z=-9.3223
	Perinatal diseases	0.0121(0.0054,0.0189)*;z=3.5050	0.0132(0.0062,0.0203)*;z=3.6882
	Birth month (cos)	-0.0007(-0.0011,-0.0003)*;z=-3.2108	-0.0007(-0.0011,-0.0002)*;z=-3.0659
	Born in the UK	0.0052(0.0039,0.0065)†;z=7.7218	0.0018(0.0004,0.0031)*;z=2.5152
	Education		-0.0051(-0.0057,-0.0044)†;z=-14.6416
	Income		-0.0127(-0.0130,-0.0124)†;z=-84.9808
	Age (years)	0.0016(0.0016,0.0017)†;z=84.5320	0.0010(0.0009,0.0010)†;z=45.1147
	Male	-0.0085(-0.0091,-0.0079)†;z=-26.8101	-0.0056(-0.0062,-0.0050)†;z=-17.0706
	Caucasian ethnicity	-0.0074(-0.0093,-0.0056)†;z=-7.7653	-0.0020(-0.0040,-0.0000)*;z=-1.9706
	Smoking	0.0263(0.0253,0.0273)†;z=50.9897	0.0192(0.0181,0.0202)†;z=35.6251
	Alcohol drinking	-0.0270(-0.0282,-0.0258)†;z=-44.5585	-0.0209(-0.0221,-0.0196)†;z=-32.2058
	Physical activity	-0.0064(-0.0067,-0.0061)†;z=-42.2411	-0.0067(-0.0070,-0.0064)†;z=-42.9813
Education			N=219,881
	Breastfed as a baby		0.1686(0.1490,0.1882)†;z=16.8596
	Maternal smoking		-0.3228(-0.3429,-0.3027)†;z=-31.4289
	Birthweight (kg)		0.0779(0.0641,0.0916)†;z=11.1100
	Perinatal diseases		-0.0426(-0.2423,0.1572);z=-0.4179
	Birth month (cos)		0.0137(0.0013,0.0262)*;z=2.1573
	Born in the UK		-0.7993(-0.8333,-0.7653)†;z=-46.0706
Income			N=194,800
	Breastfed as a baby		-0.1086(-0.1262,-0.0911)†;z=-12.1634
	Maternal smoking		0.0123(-0.0055,0.0301);z=1.3581
	Birthweight (kg)		0.1312(0.1186,0.1438)†;z=20.4590
	Perinatal diseases		-0.0675(-0.2483,0.1132);z=-0.7322
	Birth month (cos)		0.0051(-0.0062,0.0164);z=0.8909
	Born in the UK		0.0541(0.0209,0.0873)*;z=3.1929
	Education		1.2703(1.2530,1.2877)†;z=143.3220

*Significant at 0.05; † Significant at 0.0001

Generalized structural equation models: Frailty (Gaussian; identity), Education (Bernoulli, logit), Income (Ordinal, logit).

Model 1 includes early life predictors controlled with age, gender, ethnicity, smoking, alcohol drinking and physical activity covariates. Model 2 includes early life predictors controlled with age, gender, ethnicity, smoking, alcohol drinking and physical activity covariates, and education and income mediators.

For peer review only - http://bmjopen.bmj.com/site/about/guidelines.xhtml

Supplementary Table 3. Sensitivity analysis: Regression models predicting frailty index

	**Non-imputed data[1] (n=190,575)**	**Imputed data[1] (n=502,489)**
Breastfed as a baby	-0.0034(-0.0041,-0.0027)†	-0.0042(-0.0047,-0.0036)†
Maternal smoking	0.0116(0.0109,0.0123)†	0.0125(0.0120,0.0130)†
Birthweight (kg)	-0.0023(-0.0028,-0.0018)†	-0.0024(-0.0028,-0.0020)†
Perinatal diseases	0.0132(0.0062,0.0203)*	0.0093(0.0046,0.0140)†
Birth month (cos)	-0.0007(-0.0011,-0.0002)*	-0.0006(-0.0009,-0.0003)†
Born in the UK	0.0018(0.0004,0.0031)*	0.0001(-0.0008,0.0010)
Education	-0.0051(-0.0057,-0.0044)†	-0.0060(-0.0064,-0.0055)†
Income	-0.0127(-0.0130,-0.0124)†	-0.0139(-0.0141,-0.0137)†
Age (years)	0.0010(0.0009,0.0010)†	0.0009(0.0009,0.0010)†
Male	-0.0056(-0.0062,-0.0050)†	-0.0045(-0.0049,-0.0041)†
Caucasian ethnicity	-0.0020(-0.0040,-0.0001)*	-0.0015(-0.0026,-0.0004)*
Smoking	0.0192(0.0181,0.0202)†	0.0194(0.0187,0.0201)†
Alcohol drinking	-0.0209(-0.0221,-0.0196)†	-0.0203(-0.0210,-0.0195)†
Physical activity	-0.0067 (-0.0070,-0.0064)†	-0.0074(-0.0076,-0.0072)†
Intercept	0.1578(0.1542,0.1614)†	0.1672(0.1650,0.1694)†

Note: [1] presented are coefficients (95% confidence intervals); *Significant at 0.05; † Significant at 0.0001. Non-imputed analysis was based on 190,575 respondents with complete information on all variables. Maternal smoking around birth variable has 13.86% missing data, breastfed as a baby variable has 23.64% missing data, birthweight variable has 44.88% missing data, education variable has 2.02% missing data, average total household income before tax variable has 15.36% missing data, moderate or vigorous physical activity variable has 2.43% missing data. The imputed analysis included all the respondents (n=502,489).

For peer review only

For peer review only - http://bmjopen.bmj.com/site/about/guidelines.xhtml

Supplementary Table 4. Generalized structural equation models of frailty for participants who have age above 60 years old

		Model 1 Coef.(95%CI)	***Model 2*** Coef.(95%CI)
Frailty		N=73,941	N= 61,431
	Breastfed as a baby	-0.0041(-0.0054,-0.0028)†	-0.0029(-0.0043,-0.0015)†
	Maternal smoking	0.0122(0.0110,0.0134)†	0.0114(0.0101,0.0127)†
	Birthweight (kg)	-0.0022(-0.0030,-0.0014)†	-0.0020(-0.0029,-0.0012)†
	Perinatal diseases	0.0129(-0.0003,0.0261)	0.0148(0.0007,0.0290)*
	Birth month (cos)	-0.0007(-0.0014,0.0001)	-0.0006(-0.0014,0.0002)
	Born in the UK	0.0053(0.0026,0.0080)*	0.0001(-0.0029,0.0030)
	Education		-0.0069(-0.0082,-0.0056)†
	Income		-0.0111(-0.0117,-0.0105)†
	Age (years)	0.0026(0.0024,0.0028)†	0.0017(0.0015,0.0019)†
	Male	-0.0055(-0.0066,-0.0044)†	-0.0017(-0.0029,-0.0005)*
	Caucasian ethnicity	-0.0119(-0.0167,-0.0071)†	-0.0083(-0.0136,-0.0030)*
	Smoking	0.0213(0.0193,0.0234)†	0.0165(0.0143,0.0187)†
	Alcohol drinking	-0.0250(-0.0269,-0.0230)†	-0.0207(-0.0229,-0.0186)†
	Physical activity	-0.0079(-0.0085,-0.0073)†	-0.0078(-0.0084,-0.0072)†
Education			N=75,181
	Breastfed as a baby		0.1838(0.1438,0.2237)†
	Maternal smoking		-0.1563(-0.1934,-0.1193)†
	Birthweight (kg)		0.0471(0.0237,0.0704)†
	Perinatal diseases		-0.1852(-0.5974,0.2271)
	Birth month (cos)		0.0125(-0.0101,0.0352)
	Born in the UK		-0.7936(-0.8590,-0.7282)†
Income			N=62,967
	Breastfed as a baby		0.0843(0.0495,0.1191)†
	Maternal smoking		0.0403(0.0079,0.0726)*
	Birthweight (kg)		0.0919(0.0709,0.1129)†
	Perinatal diseases		-0.3311(-0.6857,0.0235)
	Birth month (cos)		0.0282(0.0080,0.0484)*
	Born in the UK		-0.1172(-0.1840,-0.0504)*
	Education		1.3530(1.3208,1.3852)†

*Significant at 0.05; † Significant at 0.0001
Generalized structural equation models: Frailty (Gaussian; identity), Education (Bernoulli, logit), Income (Ordinal, logit).
Model 1 includes early life predictors controlled with age, gender, ethnicity, smoking, alcohol drinking and physical activity covariates. Model 2 includes early life predictors controlled with age, gender, ethnicity, smoking, alcohol drinking and physical activity covariates, and education and income mediators.

For peer review only - http://bmjopen.bmj.com/site/about/guidelines.xhtml

Reporting checklist for cross sectional study.

Based on the STROBE cross sectional guidelines.

Instructions to authors

Complete this checklist by entering the page numbers from your manuscript where readers will find each of the items listed below.

Your article may not currently address all the items on the checklist. Please modify your text to include the missing information. If you are certain that an item does not apply, please write "n/a" and provide a short explanation.

Upload your completed checklist as an extra file when you submit to a journal.

In your methods section, say that you used the STROBE cross sectionalreporting guidelines, and cite them as:

von Elm E, Altman DG, Egger M, Pocock SJ, Gotzsche PC, Vandenbroucke JP. The Strengthening the Reporting of Observational Studies in Epidemiology (STROBE) Statement: guidelines for reporting observational studies.

For peer review only

		Reporting Item	Page Number
**Title and abstract**			
Title	#1a	Indicate the study's design with a commonly used term in the title or the abstract	1
Abstract	#1b	Provide in the abstract an informative and balanced summary	3

		of what was done and what was found	
**Introduction**			
Background / rationale	#2	Explain the scientific background and rationale for the investigation being reported	4
Objectives	#3	State specific objectives, including any prespecified hypotheses	6
**Methods**			
Study design	#4	Present key elements of study design early in the paper	7
Setting	#5	Describe the setting, locations, and relevant dates, including periods of recruitment, exposure, follow-up, and data collection	7
Eligibility criteria	#6a	Give the eligibility criteria, and the sources and methods of selection of participants.	7
	#7	Clearly define all outcomes, exposures, predictors, potential confounders, and effect modifiers. Give diagnostic criteria, if applicable	8-9
Data sources / measurement	#8	For each variable of interest give sources of data and details of methods of assessment (measurement). Describe comparability of assessment methods if there is more than one group. Give information separately for for exposed and unexposed groups if applicable.	9
Bias	#9	Describe any efforts to address potential sources of bias	10
Study size	#10	Explain how the study size was arrived at	8

For peer review only

Quantitative variables	#11	Explain how quantitative variables were handled in the analyses. If applicable, describe which groupings were chosen, and why	8-9
Statistical methods	#12a	Describe all statistical methods, including those used to control for confounding	9-10
Statistical methods	#12b	Describe any methods used to examine subgroups and interactions	10
Statistical methods	#12c	Explain how missing data were addressed	10
Statistical methods	#12d	If applicable, describe analytical methods taking account of sampling strategy	8
Statistical methods	#12e	Describe any sensitivity analyses	10
**Results**			
Participants	#13a	Report numbers of individuals at each stage of study—eg numbers potentially eligible, examined for eligibility, confirmed eligible, included in the study, completing follow-up, and analysed. Give information separately for for exposed and unexposed groups if applicable.	7
Participants	#13b	Give reasons for non-participation at each stage	n/a
Participants	#13c	Consider use of a flow diagram	n/a
Descriptive data	#14a	Give characteristics of study participants (eg demographic,	11

For peer review only

		clinical, social) and information on exposures and potential confounders. Give information separately for exposed and unexposed groups if applicable.	
Descriptive data	#14b	Indicate number of participants with missing data for each variable of interest	11
Outcome data	#15	Report numbers of outcome events or summary measures. Give information separately for exposed and unexposed groups if applicable.	11
Main results	#16a	Give unadjusted estimates and, if applicable, confounder-adjusted estimates and their precision (eg, 95% confidence interval). Make clear which confounders were adjusted for and why they were included	11-12
Main results	#16b	Report category boundaries when continuous variables were categorized	11-12
Main results	#16c	If relevant, consider translating estimates of relative risk into absolute risk for a meaningful time period	12
Other analyses	#17	Report other analyses done—e.g., analyses of subgroups and interactions, and sensitivity analyses	12-13
**Discussion**			
Key results	#18	Summarise key results with reference to study objectives	13
Limitations	#19	Discuss limitations of the study, taking into account sources of potential bias or imprecision. Discuss both direction and magnitude of any potential bias.	14-15

For peer review only

Interpretation	#20	Give a cautious overall interpretation considering objectives, limitations, multiplicity of analyses, results from similar studies, and other relevant evidence.	14-15
Generalisability	#21	Discuss the generalisability (external validity) of the study results	14

Other Information

Funding	#22	Give the source of funding and the role of the funders for the present study and, if applicable, for the original study on which the present article is based	16

The STROBE checklist is distributed under the terms of the Creative Commons Attribution License CC-BY. This checklist was completed on 17. September 2021 using https://www.goodreports.org/, a tool made by the EQUATOR Network in collaboration with Penelope.ai

For peer review only

BMJ Open

Pathways linking early life factors and frailty among middle-aged and older adults in England: Findings from the UK Biobank

Journal:	*BMJ Open*
Manuscript ID	bmjopen-2021-057511.R1
Article Type:	Original research
Date Submitted by the Author:	25-Jun-2022
Complete List of Authors:	Maharani, Asri; The University of Manchester, Social Work<br>Didikoglu, Altug; The University of Manchester, Division of Neuroscience & Experimental Psychology<br>O'Neill, Terence; University of Manchester, Centre for Epidemiology Versus Arthritis, Division of Musculoskeletal & Dermatological Sciences; Manchester University NHS Foundation Trust, UK & NIHR Biomedical Research Centre<br>Pendleton, Neil; The University of Manchester, Division of Neuroscience & Experimental Psychology<br>Canal, Maria; The University of Manchester, Division of Neuroscience & Experimental Psychology<br>Payton, Antony; The University of Manchester, Division of Informatics, Imaging & Data Sciences
**Primary Subject Heading**:	Geriatric medicine
Secondary Subject Heading:	Epidemiology, Public health
Keywords:	EPIDEMIOLOGY, GERIATRIC MEDICINE, SOCIAL MEDICINE

SCHOLARONE™
Manuscripts

I, the Submitting Author has the right to grant and does grant on behalf of all authors of the Work (as defined in the below author licence), an exclusive licence and/or a non-exclusive licence for contributions from authors who are: i) UK Crown employees; ii) where BMJ has agreed a CC-BY licence shall apply, and/or iii) in accordance with the terms applicable for US Federal Government officers or employees acting as part of their official duties; on a worldwide, perpetual, irrevocable, royalty-free basis to BMJ Publishing Group Ltd ("BMJ") its licensees and where the relevant Journal is co-owned by BMJ to the co-owners of the Journal, to publish the Work in this journal and any other BMJ products and to exploit all rights, as set out in our licence.

The Submitting Author accepts and understands that any supply made under these terms is made by BMJ to the Submitting Author unless you are acting as an employee on behalf of your employer or a postgraduate student of an affiliated institution which is paying any applicable article publishing charge ("APC") for Open Access articles. Where the Submitting Author wishes to make the Work available on an Open Access basis (and intends to pay the relevant APC), the terms of reuse of such Open Access shall be governed by a Creative Commons licence – details of these licences and which Creative Commons licence will apply to this Work are set out in our licence referred to above.

Other than as permitted in any relevant BMJ Author's Self Archiving Policies, I confirm this Work has not been accepted for publication elsewhere, is not being considered for publication elsewhere and does not duplicate material already published. I confirm all authors consent to publication of this Work and authorise the granting of this licence.

For peer review only

Title Page

Title:

Pathways linking early life factors and frailty among middle-aged and older adults in England: Findings from the UK Biobank

Running Title:

Early-life factors and frailty

Keywords:

UK Biobank, Early-life factors, frailty

Authors and author affiliations:

Asri Maharani, MD, PhD[1*], Altug Didikoglu, PhD[2*], Terence W O'Neill, MD, PhD[3], Neil Pendleton, MD, PhD[2], Maria Mercè Canal, PhD[2], Antony Payton, PhD[4]

1) Division of Nursing, Midwifery, and Social Work, The University of Manchester, UK

2) Division of Neuroscience & Experimental Psychology, The University of Manchester, UK

3) Centre for Epidemiology Versus Arthritis, Division of Musculoskeletal & Dermatological Sciences, The University of Manchester, UK & NIHR Biomedical Research Centre, Manchester University NHS Foundation Trust, Manchester.

4) Division of Informatics, Imaging & Data Sciences, The University of Manchester, UK

* These authors contributed equally to this work

Corresponding author's full address and corresponding author's current email:

Name: Altug Didikoglu

Address: Division of Neuroscience & Experimental Psychology, The University of Manchester, AV Hill Building, Oxford Road, M13 9PL, Manchester, UK

Email: altug.didikoglu@manchester.ac.uk

Acknowledgements

For peer review only - http://bmjopen.bmj.com/site/about/guidelines.xhtml

For peer review only

Altug Didikoglu was supported by a grant from the Republic of Turkey Ministry of National Education. This research has been conducted using the UK Biobank Resource under Application Number 41877.

Conflicts of interest

All authors declare no conflicts of interest.

Main text word counts: 3482

Number of data elements: 4

For peer review only

For peer review only - http://bmjopen.bmj.com/site/about/guidelines.xhtml

Abstract

Objectives Exposures in utero and during infancy may impact the development of diseases later in life. They may be linked with development of frailty though the mechanism is unclear. This study aims to determine the associations between early life risk factors and development of frailty amongst middle-aged and older adults as well as potential pathways via education, for any observed association.

Design A cross-sectional study.

Settings This study used data from UK Biobank, a large population-based cohort.

Participants 502,489 individuals aged 37-73 years were included in the analysis.

Primary and secondary outcome measures Early life factors in this study included being breastfed as a baby, maternal smoking, birth weight, the presence of perinatal diseases, birth month, and birth place (in or outside the UK). We developed a frailty index comprising 49 deficits. We used generalised structural equation modelling to examine the associations between early life factors and development of frailty and whether any observed association was mediated via educational attainment.

Results A history of breastfeeding and higher birth weight were associated with a lower frailty index while maternal smoking, the occurrence of perinatal diseases and birth month with a longer day length were associated with a higher frailty index. Educational level mediated the relationship between these early life factors and frailty index.

Conclusions This study highlights that biological and social risk occurring at different stages of life are related to the variations in frailty index in later life and suggests opportunities for prevention across the life course.

Keywords early life factors, frailty, generalised structural equation model, UK Biobank

For peer review only

Article Summary

Strengths and limitations of this study

- Using a large cohort of British adults in middle and older age, this study was sufficiently powered to identify associations between early life factors and frailty index.
- The findings provide the first evidence that education mediates the association between early life factors and frailty index.
- As the cohort is not nationally representative, the findings cannot be generalised to the general population.
- The questionnaire on early life factors was based on self-report and is therefore subject to recall error.

For peer review only

For peer review only - http://bmjopen.bmj.com/site/about/guidelines.xhtml

Introduction

As the world's population ages, a major goal is the attainment of increased life expectancy accompanied by fewer years spent in poor health and with disability and dependency. The worldwide population of older people (65 years and above) is predicted to double from 0.7 billion (9%) in 2019 to 1.5 billion (16%) in 2050 [1]. In addition, there is evidence that the number of disability adjusted life years (DALY) among those aged 60 years and older is increasing (from 434 million in 1990 to 574 million in 2010) [2], which will increase demand for health and care services. As physical disability is an adverse outcome of frailty [3], more research in geriatrics and gerontology has focused on defining and recognising frailty among older people with the aim of determining preventive and interventional measures [4].

Frailty can be defined as a state of increased vulnerability resulting from an age-related decline in physiologic and cognitive reserves and function following stressor events [5]. The frailty index approach, developed by Rockwood et al. [6], measures frailty level as the number of deficits presents over the number of deficits considered, including symptoms, diagnoses, disabilities, and functional impairments. Frailty has become more common with the ageing of the population. A systematic review including 240 studies from 62 countries showed that 24% of people aged 50 years and older are frail as calculated using the frailty index approach [7]. Frailty has been found to be associated with adverse health outcomes including loss of mobility, disability, falls, hospitalisation, need for long-term care, and death [8-10]. Understanding the factors that are associated with frailty is thus important for developing interventions to prevent frailty and for providing directions for future public health policies.

A growing body of literature acknowledges that the first two decades of human life are critical in determining adult life trajectories. Among the early life factors, body size at birth [11-12], cigarette smoke exposure in utero [13], infants exclusively breastfed [14], birth month [15], and the presence of perinatal diseases [16] have been found to be associated with adult chronic

For peer review only - http://bmjopen.bmj.com/site/about/guidelines.xhtml

diseases. However, the study linking those factors and frailty is limited. In addition, the evidence on the link between early life factors and occurrence of frailty have been mixed [17,18]. The present study thus aims to determine the associations between early life factors, including a history of being breastfed, maternal smoking, birth weight, the presence of perinatal diseases, birth in or outside of the UK or outside the UK and birth month, and frailty in UK adults.

Furthermore, this study contributes to the literature investigating the determinants of health in later life by exploring the pathways of early life factors that have a lasting impact on health in middle and old age. The pathway hypothesis posits that early life conditions are important not only because they are directly associated with late life but also because they shape later life experiences [19,20], including restricted educational attainment and life chances. The most frequently hypothesised pathway between circumstances in early stages of life and adult health is adult socioeconomic status. Pakpahan et al. showed that socioeconomic factors in adulthood, including education, mediate the link between childhood health and socioeconomic conditions and self-rated health among older Europeans [19]. Because interventions that target common pathways have the potential to reduce frailty, the identification of the pathways of early life factors leading to frailty later in life has substantial public health relevance for the translation of life course epidemiology into practice. The present study considers whether any observed association between early life factors and frailty could be attributed to differences in education attainment (Figure 1).

Methods

Source and Sample

Data were drawn from the UK Biobank, a prospective cohort study of the genetic, environmental and lifestyle causes of diseases among adults in the UK [21]. The study involved the collection of extensive questionnaire data and biological samples from, and the

For peer review only - http://bmjopen.bmj.com/site/about/guidelines.xhtml

performance of, physical examinations of more than 500,000 respondents enrolled at 22 assessment sites in England, Scotland, and Wales between 2006 and 2010. Subjects who took part provided written informed consent for data collection, analysis and linkage; they also completed a touchscreen questionnaire, a nurse-led interview, and had their physical measurements taken. The UK Biobank invited adults who were registered with a general practitioner and who lived within reasonable traveling distance of the assessment centre. The current study includes 502,489 individuals aged 37-73 years who had study-specific available data and were not withdrawn from the study. This study was conducted as part of UK Biobank Project Number 41877 and is covered by the generic ethics approval for UK Biobank studies from the NHS National Research Ethics Service (16/NW/0274).

Measures

Early life factors

Information by questionnaire was obtained on: maternal smoking in the pre- and post-natal period, history of being breastfed as a baby, birth month, birthweight, the presence of perinatal diseases, and place of birth. We defined maternal smoking based on the question 'Did your mother smoke regularly around the time when you were born?' (Data-Field 1787). Respondents were categorised as having been breastfed as babies if they answered 'yes' to the question: 'Were you breastfed when you were a baby?' (Data-Field 1677). We retrieved information on birth month from the birth date (Data-Field 52) and treated it as the cosine of the values, representing the rhythmic seasonal length of day and night. We considered this might represent daylight time better than treating it as a categorical variable. This is an approach which we have used in a previous study [22]. Birth months of participants born in the UK and other countries in the southern hemisphere were converted to their antiphase. Information on birthweight was gathered by means of self-reported birthweight in kilograms (Data-Field 20022). We categorised the birth weight into low birth weight (<2,500 g), normal

For peer review only

birth weight (2,500 – 4,000), and high birth weight (>4,000 gr). The presence of perinatal diseases (‘ICD10 Chapter XVI: Certain conditions originating in the perinatal period’) was coded as one based on self-reported medical history (Category 2416). We categorised the place birth of the respondents as born in the UK or outside the UK (Data-Field 1647). Answers of ‘Do not know’ or ‘Prefer not to answer’ were accepted as missing for all questions.

Education

The education variable represents the highest educational level completed by the respondents. Qualifications were categorised as high school or less (reference) and college or university degree (Data-Field 6138).

Frailty index

Following William et al. [23], we derived the frailty index using 49 functional, psychological, and social deficits within the range of data variables in the UK Biobank (see Supplementary Table 1). We coded the binary variables as 0 or 1, and for ordinal and continuous variables, coding was based on distribution. The total number of deficits was summed and divided by total possible deficits to create a frailty index between 0 and 1, where higher scores indicated greater frailty.

Covariates

We included demographic and health behaviour as covariates. Demographic information included age (in years; Data-Field 21003), gender (with male as the reference; Data-Field 31), and ethnicity (other than Caucasian as the reference or Caucasian; Data-Field 21000). Health behaviours included physical activity, alcohol intake and smoking status. Physical activity was measured as the number of days per week respondents engaged in at least 10 minutes of moderate or vigorous physical activity (Data-Field 884, Data-Field 904). Respondents were classified as non-current smokers (reference) or current smokers (Data-Field 20116). Alcohol

For peer review only - http://bmjopen.bmj.com/site/about/guidelines.xhtml

intake status was classified as non-current (reference) or current alcohol drinking (Data-Field 20117).

Statistical Analyses

Descriptive statistics were used to summarise subject characteristics including means and standard deviation for continuous variables and frequencies and percentages for categorical variables. We looked at the associations between frailty index and both early life factors and other covariates using unpaired t-tests (dichotomous variables), ANOVA (categorical variables), and Pearson's correlation (continuous variables).

We first performed a multivariate regression model including early life factors, education, and covariates (age, gender, ethnicity, smoking, alcohol drinking, physical activity). We further handled missing data using multivariate imputation by chained equations (MICE) [24] (using Stata's mi program) [25]. Twenty imputations were used.

The structural equation model (SEM) has been widely used to investigate complex relationships between variables in epidemiological studies [26]. SEM can be used to resolve the endogeneity problem between variables and to explore direct, indirect, and total effects between exogenous and endogenous variables. It can jointly test a variety of hypotheses that involve different types of complicated cause-effect relationships. However, all responses are assumed to be continuous, even when a variable is binary or categorical. In our analysis we include binary (education). To address this, we used a generalised structural equation model (GSEM) to identify the link between early life factors and frailty index and the mediating effect of education and income on that relationship. A GSEM combines generalised linear model (GLM) estimation and SEM modelling estimation; it can accommodate binary, ordinal, counted and categorical data [27]. Using maximum likelihood estimators, GLM estimators are based on a density function, allowing the direct use of all types of data [28]. The analyses were performed using MPlus version 8. We examined education as mediators of the

For peer review only - http://bmjopen.bmj.com/site/about/guidelines.xhtml

relationship in the GSEM model, which were controlled for age, gender, ethnicity and health behaviours (Model fit information: Chi-square=5049.35, p value=0.00; RMSEA=0.06, CFI=0.82; WRMR=13.01).

Patient and public involvement

Patients and/or the public were not involved in this study.

Results

Subjects

The study sample consisted of 502,489 respondents with an average age of 56.53 years (standard deviation [SD]=8.10 years) (Table 1). Just under half (45%) of the respondents were male, and most were Caucasian (94.59%). Around one-third of the respondents had graduated from college or university. The proportion of respondents whose mothers smoked regularly around the time of their birth was 29%. More than 72% of respondents were breastfed as babies, and 0.18% had perinatal diseases. 10% of respondents had low birth weight, while 13% of them had high birth weight. 91% of the respondents were born in the UK. Just over two-thirds of subjects reported engaging in at least 10 minutes of moderate or vigorous physical activity at least three days per week; 92% consumed alcohol and 11% were current smokers.

Early life factors, covariates and frailty index

In bivariate analyses, compared to those whose mothers did not smoke around birth, maternal pre- and post-natal smoking was associated with a significantly higher frailty index (0.146 vs 0.133) as was the presence of perinatal diseases (0.149 vs 0.138) and being born in the UK (0.138 vs 0.137). A history of breast feeding was associated with a lower frailty index (0.134 vs 0.137). Low (0.149 vs 0.131) and high (0.136 vs 0.138) birthweight were associated with higher frailty scores compared to normal birthweight. Shorter daylight hours at birth (r=-0.01) were associated with lower frailty indices. As expected, the frailty index was higher among

For peer review only - http://bmjopen.bmj.com/site/about/guidelines.xhtml

women than among men and in those with lower educational attainment. The frailty index was also higher in smokers, non-drinkers and those who engaged in less physical activity.

In regression analyses the effects of early life factors and covariates on the frailty index appeared similar in terms of both magnitude and direction when using both non-imputed and imputed data (see Supplementary Table 2). In these multivariate regression analyses adjusting for age, gender and health behaviours, birth month with longer hours of daylight, having a low and high birthweight, maternal smoking, being breastfed as baby, perinatal diseases and born in the UK had positive and significant associations with frailty index.

Mediation analysis

In the GSEM model, education mediated the association between early life factors and frailty index among middle-aged and older adults, supporting the pathway hypothesis. Table 2 presents the total, direct and indirect effects for each of the early life factor on the frailty index. Maternal smoking (direct effect: coef.=0.068, z=33.40; indirect effect: coef.=0.011, z=25.54) and low (direct effect: coef.=0.041, z=20.93; indirect effect: coef.=0.003, z=9.18) and high birthweight (direct effect: coef.=0.013, z=6.34; indirect effect: coef.=0.001, z=4.09) were directly and indirectly affecting frailty index compared to normal birthweight. The direct and indirect effects of breastfed as a baby on lower frailty index were -0.022 (z=-10.36) and -0.009 (z=-22.91). Perinatal diseases had significant direct effect on higher frailty index (coef.=0.007, z=3.83), but it had no indirect effect on the frailty index (coef.=0.000, z=0.27). Born in the UK, differently, had significant indirect effect on higher frailty index (coef.=0.016, z=31.24), but it had no direct effect on the frailty index (coef.=0.002, z=0.74). Birth months with short daylight was affecting lower frailty scores with the lowest effect size both directly (coef.=-0.006, z=-2.91) and indirectly (coef.=-0.001, p-value=-2.35).

Education mediated the links between early life factors and frailty index (Figure 2). Participants born in the UK had a lower probability of completing higher education (coef.=-

For peer review only - http://bmjopen.bmj.com/site/about/guidelines.xhtml

0.130, z=-44.65). Having been breastfed as a baby (coef.=0.076, z=26.87) were associated with higher educational attainment, while maternal smoking was associated with lower educational attainment (coef.=-0.087, z=-31.41). Had low (coef.=-0.027, z=-9.39) and high birthweight (coef.=-0.011, z=-4.11) was related to lower education attainment compared to normal birthweight. Birth months with short daylight was related to higher education with the lowest effect size (coef.=0.006, z=2.35). Higher education was also associated with a lower frailty index (coef.=-0.123, z=-44.30). Amongst covariates with greater effect sizes, older age (coef.=0.178, z=83.25), lower activity levels (coef.=-0.088, z=-45.61) and smoking (coef.=0.106, z=56.36) were associated with a higher frailty index. Drinking alcohol is related to lower frailty index (coef.=-0.093; z=-51.63).

Discussions

Using data from UK Biobank we found that a history of breastfeeding was associated with a lower frailty index, while maternal smoking, having low or high birth weight, perinatal diseases and birth month with longer day length were associated with a higher frailty index. This study provides the first evidence that educational attainment level mediates the association between early life factors and frailty index.

Early life factors have previously been linked with higher frailty and chronic disease risk later in life [29,30]. Our findings highlight the importance of early life factors in determining frailty in middle age and older individuals. Maternal smoking was directly associated with higher frailty compared to those who were not exposed to maternal smoking. Evidence has suggested that cigarette smoke exposure in utero is linked to the development of chronic diseases later in life, including type 2 diabetes, obesity, certain cancers and respiratory disorders [13]. We also showed that this association was mediated by educational attainment. This is in line with a previous study which reported lower academic achievements of adolescents whose mothers

For peer review only - http://bmjopen.bmj.com/site/about/guidelines.xhtml

smoked during pregnancy [31]. Maternal smoking during pregnancy was also found to be correlated with the children's cognitive function [32].

There is some evidence of a link between early life factors and occurrence of frailty. In a recent study in Finland, greater weight, length and BMI at birth were associated with a lower risk of frailty later in life [17]. Having low or high birth weight were associated with higher frailty index compared to had normal birthweight, both directly and indirectly through education. Bleker and colleagues found that prenatal undernutrition was not associated with frailty but was associated with poorer health in old age, including slower gait speed and lower physical functioning and the findings remained significant after inclusion of an extensive set of control variables including adult socioeconomic status [18]. Low birth weight is associated with increased risk of age-related diseases in prior review, and insulin-like growth factor (IGF-1) is the key driver of this process [33]. High birth weight may be the results of maternal obesity,[34] and a study in Finland found that being born large for gestational age at term was associated with thicker carotid intima medial as the marker of subclinical atherosclerosis [35]. We also found that individuals who reported that they were breastfed have lower frailty score. Infants exclusively breastfed have been found to have a lower risk of obesity, type 2 diabetes and high blood pressure in adulthood [14].

Birth moths is associated with lower frailty index with a limited effect sizes in our study. In a large study in the US with 1,749,400 individuals showed that spring summer-born individual have relatively higher risk than autumn-winter born individuals and these seasons coincide with lower life expectancy [36]. This study showed that not only cardiovascular diseases but also several chronic diseases were found associated with season of birth with having a different seasonal pattern. The underlying mechanisms may differ for each of these associations such as sensitization to allergens or vitamin D deficiency [36]. Another possible mechanism is that differential light exposure during perinatal period influences development

For peer review only - http://bmjopen.bmj.com/site/about/guidelines.xhtml

of the biological clock, in turn influencing later-life circadian rhythms and sleep system which are essential for health [37]. In European countries, it was shown that spring/summer born participants compared to autumn had higher frailty score but this effect seemed independent of education [38]. However, we found and indirect effect of season of birth through education. the indirect relationship of season of birth and frailty may be due to social factors such as September date cut-off to determine age when they start education, which is in line with our findings showing an association between winter-born individuals and higher education [39,40].

Our results further suggest that had a perinatal disease was associated directly with higher frailty index. New-borns' perinatal complications are related to accelerated ageing at midlife [16]. Being born in the UK only affecting the frailty index indirectly through education, but not directly. Respondents who were not born in the UK were likely to have higher education attainment, which in turn able to better maintain their health during in later stage of their lives. However, we should note that our sample in this this analysis may not be the representation of general population and that participants were categorised as being born outside UK without taking into account the country of origin and their background. In our analysis we observed that education levels mediate the link between the other early life factors and the frailty index. Early life factors have a significant relationship with educational attainment, and higher education attainment is linked to lower frailty index. This result is broadly in keeping with a prior study in Sweden which found that the associations between childhood conditions and various old age health indicators (musculoskeletal disorders, cardiovascular disease, self-rated health and impaired mobility) are mediated by education [41]. Prior research on the biological and psychological pathways linked childhood health and socioeconomic conditions to self-reported health status among older adults in 15 European countries [19]. Early life health is marked by developmental plasticity; life-course trajectories of socioeconomic

For peer review only - http://bmjopen.bmj.com/site/about/guidelines.xhtml

attainment could be altered by physical and social conditions [42] and set cascading physiological processes in motion, impacting health decades later [43]. Our findings have potential implications for policies aiming at preventing frailty among older adults. Subsequent circumstances mediate the impact of early life factors on frailty later in life, and our study suggests that interventions such as improving education in midlife may mitigate early life disadvantages.

Our findings are based on a large and well characterised cohort. There are, however, a number of limitations to be consider in interpreting the results. First, information concerning early life factors in this study was based on self-report and is therefore subject to recall error. The likely effect of such error would be to underestimate the relationship between these factors and the frailty index. Second, we have limited access to the health conditions of the parents. A broad range of conditions which are comprised in the frailty index bear a hereditary risk, thus taking into account the health conditions of the parents is important in assessing the independent associations with frailty. Future studies may include the health conditions of the parents as the covariates. Third, the information on breast feeding duration is unavailable. Breast feeding for weeks rather than months may confer different outcomes. A dose response relationship is thus cannot be assessed. Finally, the data were based on a sample of predominantly Caucasian men and women and should be extrapolated beyond this group with caution [44].

In conclusion, this study indicates an association between early life factors and frailty later in life. Early life conditions are important as the start of a mediated, incremental process during the life course. A comprehensive understanding of the determinants of frailty among middle-aged and older adults requires attention to exposures throughout the entire life course, with a special focus on the in utero and infancy stages and the chains of associated socioeconomic conditions that that connect over the life course. Applying a life course perspective to health in adulthood and old age should have implications for public health interventions, social

For peer review only

For peer review only - http://bmjopen.bmj.com/site/about/guidelines.xhtml

policy, and further research. Early life is not the only period for any potential successful intervention; as our findings show, early life disadvantages may be offset by education and material wealth. Interventions throughout the life course, and especially during early life, could substantially reduce the health burden later in life.

For peer review only

For peer review only - http://bmjopen.bmj.com/site/about/guidelines.xhtml

Contributorship statement

A.D and A.M performed the data analysis. A.D and A.M drafted the manuscript. N.P, T.W.O, M.M.C., A.P. and were involved in planning and supervised the work. All authors discussed the results and commented on the manuscript.

Competing interest

All authors declare no conflicts of interest.

Funding

Altug Didikoglu was supported by a grant from the Republic of Turkey Ministry of National Education. This research has been conducted using the UK Biobank Resource under Application Number 41877.

Data sharing statement

Data are available in a public, open access repository. Other researchers can apply for UK Biobank data to answer specific research questions.

For peer review only

References

1. United Nations. World Population Prospects 2019: Highlights. New York: Department of Economic and Social Affairs; 2019. Contract No.: (ST/ESA/SER.A/423).
2. Prince MJ, Wu F, Guo Y, et al. The burden of disease in older people and implications for health policy and practice. *Lancet*. 2015;385:549-562.
3. Sternberg SA, Schwartz AW, Karunananthan S, Bergman H, Clarfield MA. The identification of frailty: a systematic literature review. *J Am Geriatr Soc*. 2011;59:2129-2138.
4. Daniels R, van Rossum E, de Witte L, Kempen GI, van den Heuvel W. Interventions to prevent disability in frail community-dwelling elderly: a systematic review. *BMC Health Serv Res*. 2008;8:278.
5. Hogan DB. Models, definitions, and criteria for frailty. *Conn's Handbook of Models for Human Aging*. 2018:35-44.
6. Rockwood K, Mitnitski A. Frailty defined by deficit accumulation and geriatric medicine defined by frailty. *Clin Geriatr Med*. 2011;27:17-26.
7. O'Caoimh R, Sezgin D, O'Donovan MR, et al. Prevalence of frailty in 62 countries across the world: a systematic review and meta-analysis of population-level studies. *Age and Ageing*. 2021;50:96-104.
8. Hanlon P, Nicholl BI, Jani BD, Lee D, McQueenie R, Mair FS. Frailty and prefrailty in middle-aged and older adults and its association with multimorbidity and mortality: a prospective analysis of 493 737 UK Biobank participants. *Lancet Public Health*. 2018;3:e323-e332.
9. Cegri F, Orfila F, Abellana RM, Pastor-Valero M. The Impact of Frailty on Admission to Home Care Services And Nursing Homes: Eight-Year Follow-Up of A Community-Dwelling, Older Adult, Spanish Cohort. *BMC Geriatrics*. 2020;20:1-13.
10. Ensrud KE, Ewing SK, Taylor BC, et al. Frailty and risk of falls, fracture, and mortality in older women: the study of osteoporotic fractures. *J Gerontol A Biol Sci Med Sci*. 2007;62:744-51.
11. Barker DJ. Fetal origins of coronary heart disease. *BMJ*. 1995;311:171-4.
12. Eriksson JG. Developmental origins of health and disease–from a small body size at birth to epigenetics. *Ann Med*. 2016;48:456-467.

For peer review only

For peer review only - http://bmjopen.bmj.com/site/about/guidelines.xhtml

13. Doherty S, Grabowski J, Hoffman C, Ng S, Zelikoff J. Early life insult from cigarette smoke may be predictive of chronic diseases later in life. *Biomarkers*. 2009;14(sup1):97-101.
14. Horta BL, Loret de Mola C, Victora CG. Long-term consequences of breastfeeding on cholesterol, obesity, systolic blood pressure and type 2 diabetes: a systematic review and meta-analysis. *Acta Paediatr*. 2015;104:30-7.
15. Zhang Y, Devore EE, Strohmaier S, Grodstein F, Schernhammer ES. Birth month, birth season, and overall and cardiovascular disease mortality in US women: prospective cohort study. *BMJ*. 2019;367.
16. Shalev I, Caspi A, Ambler A, et al. Perinatal complications and aging indicators by midlife. *Pediatrics*. 2014;134:e1315-e23.
17. Haapanen M, Perälä M, Salonen M, et al. Early life determinants of frailty in old age: the Helsinki Birth Cohort Study. *Age and Ageing*. 2018;47:569-75.
18. Bleker LS, de Rooij SR, Painter RC, van der Velde N, Roseboom TJ. Prenatal undernutrition and physical function and frailty at the age of 68 years: the Dutch Famine Birth Cohort Study. *J Gerontol A Biol Sci Med Sci*. 2016;71:1306-14.
19. Pakpahan E, Hoffmann R, Kröger H. The long arm of childhood circumstances on health in old age: Evidence from SHARELIFE. *Advances in Life Course Research*. 2017;31:1-10.
20. Power C, Hertzman C. Social and biological pathways linking early life and adult disease. *Br Med Bull*. 1997;53(1):210-21.
21. Sudlow C, Gallacher J, Allen N, Beral V, Burton P, Danesh J, et al. UK biobank: an open access resource for identifying the causes of a wide range of complex diseases of middle and old age. *PLoS Medicine*. 2015;12(3):e1001779.
22. Didikoglu A, Maharani A, Pendleton N, Canal MM, Payton A. Early life factors and COVID-19 infection in England: A prospective analysis of UK Biobank participants. *Early Human Development*. 2021;155:105326.
23. Williams DM, Jylhävä J, Pedersen NL, Hägg S. A frailty index for UK Biobank participants. *J Gerontol A Biol Sci Med Sci*. 2019;74(4):582-7.
24. Azur MJ, Stuart EA, Frangakis C, Leaf PJ. Multiple imputation by chained equations: what is it and how does it work? *International Journal of Methods in Psychiatric Research*. 2011;20(1):40-9.
25. StataCorp. Stata Multiple-Imputation Reference Manual Release 17. College Station, TX: StataCorp LLC; 2021.

For peer review only - http://bmjopen.bmj.com/site/about/guidelines.xhtml

26. Amorim LDAF, Fiaccone RL, Santos CAS, Santos TNd, de Moraes LTL, Oliveira NF, et al. Structural Equation Modeling in Epidemiology. *Cad Saúde Pública*. 2010;26(12):2251-62.
27. Zhang L, Zhang J. Perception of small tourism enterprises in Lao PDR regarding social sustainability under the influence of social network. *Tourism Management*. 2018;69:109-20.
28. McCullagh P, Nelder JA. *Generalized Linear Models*: Routledge; 2019.
29. Michels KB. Early life predictors of chronic disease. *J Womens Health*. 2003;12(2):157-61.
30. Li Y, Xue Q-L, Odden MC, Chen X, Wu C. Linking early life risk factors to frailty in old age: evidence from the China Health and Retirement Longitudinal Study. *Age and Ageing*. 2020;49(2):208-17.
31. Kristjansson AL, Thomas S, Lilly CL, Thorisdottir IE, Allegrante JP, Sigfusdottir ID. Maternal smoking during pregnancy and academic achievement of offspring over time: a registry data-based cohort study. *Preventive Medicine*. 2018;113:74-9.
32. Julvez J, Ribas-Fitó N, Torrent M, Forns M, Garcia-Esteban R, Sunyer J. Maternal smoking habits and cognitive development of children at age 4 years in a population-based birth cohort. *International Journal of Epidemiology*. 2007;36(4):825-32.
33. Vaiserman AM. Birth weight predicts aging trajectory: A hypothesis. *Mechanisms of Ageing and Development*. 2018;173:61-70.
34. Hanson M, Cooper C, Aihie Sayer A, Eendebak R, Clough G, Beard J. Developmental aspects of a life course approach to healthy ageing. *The Journal of Physiology*. 2016;594(8):2147-60.
35. Skilton MR, Viikari JS, Juonala M, Laitinen T, Lehtimäki T, Taittonen L, et al. Fetal growth and preterm birth influence cardiovascular risk factors and arterial health in young adults: the Cardiovascular Risk in Young Finns Study. *Arteriosclerosis, Thrombosis, and Vascular Biology*. 2011;31(12):2975-81.
36. Boland MR, Shahn Z, Madigan D, Hripcsak G, Tatonetti NP. Birth month affects lifetime disease risk: a phenome-wide method. *Journal of the American Medical Informatics Association*. 2015;22(5):1042-53.
37. Didikoglu A, Nair N, Robinson AC, Roncaroli F, Pendleton N, Payton A, et al. The effect of season of birth on brain epigenome-wide DNA methylation of older adults. *Journal of Developmental Origins of Health and Disease*. 2022;13(3):367-77.

For peer review only - http://bmjopen.bmj.com/site/about/guidelines.xhtml

38. Abeliansky AL, Strulik H. Season of birth, health and aging. *Economics & Human Biology*. 2020;36:100812.
39. Verachtert P, De Fraine B, Onghena P, Ghesquière P. Season of birth and school success in the early years of primary education. *Oxford Review of Education*. 2010;36(3):285-306.
40. Department of Education. Month of Birth and Education. Schools Analysis and Research Division. 2010. Contract No.: Research Report DFE-RR017.
41. Berndt H, Fors S. Childhood living conditions, education and health among the oldest old in Sweden. *Ageing & Society*. 2016;36(3):631-48.
42. Haas SA, Glymour MM, Berkman LF. Childhood health and labor market inequality over the life course. *Journal of Health and Social Behavior*. 2011;52(3):298-313.
43. Montez JK, Hayward MD. *Early life conditions and later life mortality*. International Handbook of Adult Mortality: Springer; 2011. p. 187-206.
44. Fry A, Littlejohns TJ, Sudlow C, Doherty N, Adamska L, Sprosen T, et al. Comparison of sociodemographic and health-related characteristics of UK Biobank participants with those of the general population. *American Journal of Epidemiology*. 2017;186(9):1026-34.

List of the figures and tables

Figure 1 The pathways of early life factors and impact on frailty among adults

Figure 2 Generalised structural equation models to identify the association between early life factors and frailty index, and education as mediators of the relationship between early life factors and frailty index. Note: *Significant at 0.05; † Significant at 0.0001

Table 1 Subject characteristics

Table 2 Total, direct, and indirect effects of early life factors on frailty index

For peer review only

Table 1. Subject characteristics (n=502,489)

Variable	Percentage or mean (SD)*	Mean (SD) of frailty index**	Bivariate association with frailty index***
Frailty index, mean (SD)	0.14(0.08)		
Early-life factors			
Maternal smoking around birth, %			p<0.0001
No	70.75%	0.133(0.073)	
Yes	29.25%	0.146(0.078)	
Breastfed as a baby, %			p<0.0001
No	27.65%	0.137(0.076)	
Yes	72.35%	0.134(0.074)	
Birthweight, %			p<0.0001
Low birth weight	10.26%	0.149(0.080)	
Normal birth weight	76.34%	0.131(0.073)	
High birth weight	13.40%	0.136(0.076)	
Birth month, %			p=0.0002
January	8.44%	0.138(0.076)	
February	7.96%	0.137(0.075)	
March	8.98%	0.138(0.075)	
April	8.59%	0.139(0.076)	
May	8.98%	0.138(0.076)	
June	8.45%	0.139(0.076)	
July	8.48%	0.139(0.076)	

For peer review only

August	8.24%	0.138(0.076)	
September	8.14%	0.138(0.075)	
October	8.06%	0.137(0.076)	
November	7.63%	0.137(0.075)	
December	8.03%	0.138(0.076)	
Perinatal diseases, %			p<0.0001
No	99.82%	0.138(0.075)	
Yes	0.18%	0.149(0.084)	
Born in the UK, %			p=0.0381
No	8.96%	0.137(0.076)	
Yes	91.04%	0.138(0.075)	
Sociodemographics			
Age (years), mean (SD)	56.53(8.10)		R=0.16, p<0.0001
Gender, %			p<0.0001
Female	54.40%	0.141(0.075)	
Male	45.60%	0.134(0.076)	
Ethnicity, %			p<0.0001
Other	5.41%	0.141(0.078)	
Caucasian	94.59%	0.138(0.075)	
Education, %			p<0.0001
Less than college	67.27%	0.145(0.077)	
College or university degree	32.73%	0.122 (0.069)	
Health behaviours			

For peer review only - http://bmjopen.bmj.com/site/about/guidelines.xhtml

Moderate or vigorous physical activity, %			p<0.0001
None	10.75%	0.160(0.085)	
1 day	7.11%	0.134(0.072)	
2 days	13.40%	0.133(0.072)	
3 days or more	68.75%	0.135(0.073)	
Current alcohol consumption, %			p<0.0001
No	8.08%	0.166(0.088)	
Yes	91.92%	0.135(0.074)	
Current smoking, %			p<0.0001
No	89.39%	0.135(0.074)	
Yes	10.61%	0.159(0.084)	

Note: * Presented are means (standard deviation) for continuous variables and percentages for categorical variables. The maternal smoking variable includes 13.86% missing data, the breastfed as a baby variable includes 23.64% missing data, the birthweight variable includes 44.88% missing data, the education variable includes 2.02% missing data, and the moderate or vigorous physical activity variable includes 2.43% missing data. ** Presented are the means (standard deviation) of the frailty index per group. *** Bivariate analyses are unpaired t-tests for binary variables, ANOVA for ordinal variables, and Pearson's correlation for continuous variables.

For peer review only - http://bmjopen.bmj.com/site/about/guidelines.xhtml

Table 2. Total, direct, and indirect effects of early life factors on frailty index

	Total effects	Direct effects	Indirect effects
Breastfed as a baby	-0.031 (0.002)†	-0.022 (0.002)†	-0.009 (0.000)†
Maternal smoking around birth	0.079 (0.002)†	0.068 (0.002)†	0.011 (0.000)†
Low birth weight	0.045 (0.002)†	0.041 (0.002)†	0.003 (0.000)†
High birth weight	0.015 (0.002)†	0.013 (0.002)†	0.001 (0.000)†
Birth month (cos)	-0.007 (0.002)*	-0.006 (0.002)*	-0.001 (0.000)*
Perinatal diseases	0.007 (0.002)†	0.007 (0.002)†	0.000 (0.000)
Born in the UK	0.018 (0.002)†	0.002 (0.002)	0.016 (0.001)†

For peer review only

For peer review only - http://bmjopen.bmj.com/site/about/guidelines.xhtml

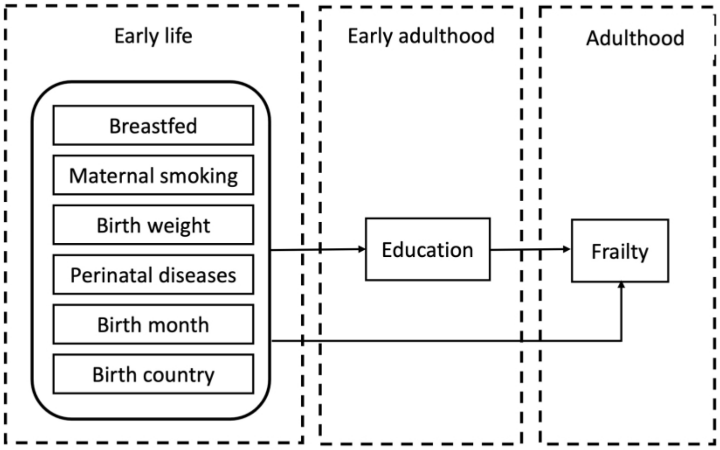

The pathways of early life factors and impact on frailty among adults

274x190mm (277 x 277 DPI)

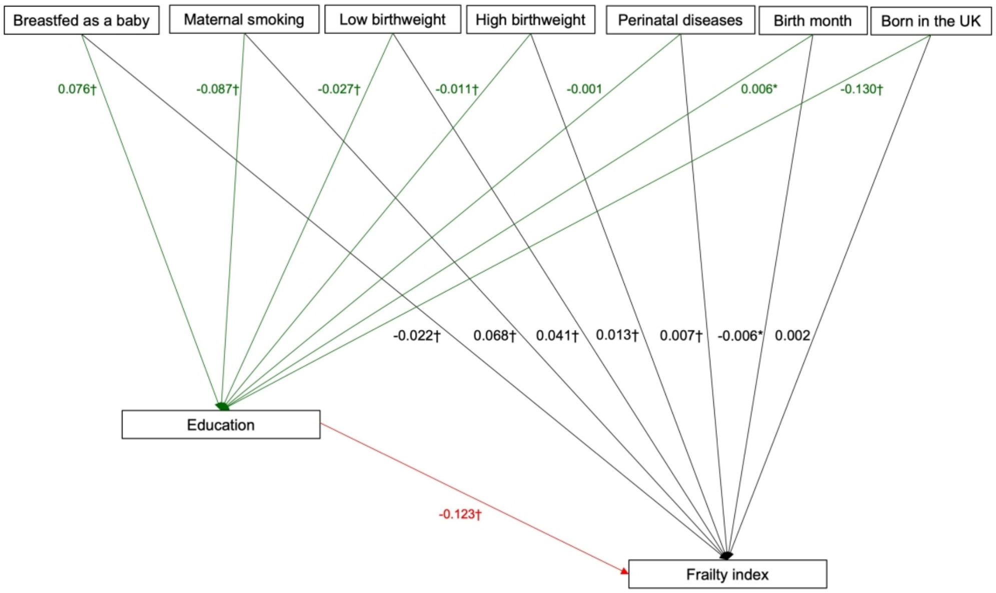

Generalised structural equation models to identify the association between early life factors and frailty index, and education as mediators of the relationship between early life factors and frailty index. Note: *Significant at 0.05; † Significant at 0.0001

274x190mm (277 x 277 DPI)

Supplementary Material: Pathways linking early life factors and frailty among middle-aged and older adults in England: Findings from UK Biobank

Supplementary Table 1. Variables included in the UK Biobank frailty indices

Item	Variable	Definition	Coding
	Sensory		
1	Glaucoma	Self-report of physician-diagnosed glaucoma	0=no; 1=yes
2	Cataracts	Self-report of physician-diagnosed glaucoma	0=no; 1=yes
3	Hearing difficulty	Self-report experiencing hearing difficulty	0=no; 1=yes/completely deaf
	Cranial		
4	Migraine	Self-report of physician-diagnosed migraine	0=no; 1=yes
5	Dental problems	Self-report of physician-diagnosed dental problems, i.e., ulcers, painful gums, bleeding gums, loose teeth, toothache, dentures	0=none; 1=any
	Mental well-being		
6	Self-rated health	Self-rated health in 4 Likert scale	0=excellent; 0.25=good; 0.5=fair; 1=poor
7	Fatigue	Self-report of frequency of tiredness / lethargy in last two weeks	0=not at all; 0.25=several days; 0.5=more than half; 1=nearly every day
8	Sleep	Self-report experiencing of sleeplessness/ insomnia	0=never/rarely; 0.5=sometimes; 1=usually
9	Depressed feelings	Self-report of frequency having depressed feeling in last two weeks	0=not at all; 0.5=several days; 0.75=more than half; 1=nearly every day
10	Self-described nervous personality	Self-report of having nervous personality	0=no; 1=yes
11	Severe anxiety/ panic attacks	Self-report of physician-diagnosed severe anxiety/panic attacks	0=no; 1=yes
12	Common to feel loneliness	Self-report of feeling lonely commonly	0=no; 1=yes
13	Sense of misery (ever/never)	Self-report of ever having sense of misery	0=no; 1=yes
	Infirmity		
14	Infirmity	Self-report of having long-standing illness or disability	0=no; 1=yes
15	Falls in last year	Self-report of experiencing falls last year	0=no falls; 0.5=one fall; 1=more than one fall
16	Fractures/broken bones in last five years	Self-report of experiencing fractures/broken bones in last five years	0=no; 1=yes
	Cardiometabolic		
17	Diabetes	Self-report of physician-diagnosed diabetes	0=no; 1=yes
18	Myocardial infarction	Self-report of physician-diagnosed myocardial infarction	0=no; 1=yes
19	Angina	Self-report of physician-diagnosed angina	0=no; 1=yes
20	Stroke	Self-report of physician-diagnosed stroke	0=no; 1=yes
21	High blood pressure	Self-report of physician-diagnosed high blood pressure	0=no; 1=yes
22	Hypothyroidism	Self-report of physician-diagnosed hypothyroidism	0=no; 1=yes
23	Deep-vein thrombosis	Self-report of physician-diagnosed deep-vein thrombosis	0=no; 1=yes
24	High cholesterol	Self-report of physician-diagnosed high cholesterol	0=no; 1=yes

For peer review only - http://bmjopen.bmj.com/site/about/guidelines.xhtml

	Respiratory		
25	Breathing	Self-report of having wheeze in last year	0=no; 1=yes
26	Pneumonia	Self-report of physician-diagnosed pneumonia	0=no; 1=yes
27	Chronic bronchitis/emphysema	Self-report of physician-diagnosed chronic bronchitis/emphysema	0=no; 1=yes
28	Asthma	Self-report of physician-diagnosed asthma	0=no; 1=yes
	Musculoskeletal		
29	Rheumatoid arthritis	Self-report of physician-diagnosed rheumatoid arthritis	0=no; 1=yes
30	Osteoarthritis	Self-report of physician-diagnosed osteoarthritis	0=no; 1=yes
31	Gout	Self-report of physician-diagnosed gout	0=no; 1=yes
32	Osteoporosis	Self-report of physician-diagnosed osteoporosis	0=no; 1=yes
	Immunological		
33	Hay fever, allergic rhinitis or eczema	Self-report of physician-diagnosed hay fever, allergic rhinitis or eczema	0=no; 1=yes
34	Psoriasis	Self-report of physician-diagnosed psoriasis	0=no; 1=yes
	Cancer		
35	Any cancer diagnosis	Self-report of physician-diagnosed any cancer	0=no; 1=yes
36	Multiple cancers diagnosed (number reported)	Self-report of physician-diagnosed multiple cancer	0=no cancer or single cancer; 1=multiple cancer
	Pain		
37	Chest pain	Self-report of ever experiencing chest pain	0=no; 1=yes
38	Head and/or neck pain	Self-report of ever experiencing head and/or neck pain	0=no; 1=yes
39	Back pain	Self-report of ever experiencing back pain	0=no; 1=yes
40	Stomach/abdominal pain	Self-report of ever experiencing stomach/abdominal pain	0=no; 1=yes
41	Hip pain	Self-report of ever experiencing hip pain	0=no; 1=yes
42	Knee pain	Self-report of ever experiencing knee pain	0=no; 1=yes
43	Whole-body pain	Self-report of ever experiencing whole-body pain	0=no; 1=yes
44	Facial pain	Self-report of ever experiencing facial pain	0=no; 1=yes
45	Sciatica	Self-report of physician-diagnosed sciatica	0=no; 1=yes
	Gastrointestinal		
46	Gastric reflux	Self-report of physician-diagnosed gastric reflux	0=no; 1=yes
47	Hiatus hernia	Self-report of physician-diagnosed hiatus hernia	0=no; 1=yes
48	Gall stones	Self-report of physician-diagnosed gall stones	0=no; 1=yes
49	Diverticulitis	Self-report of physician-diagnosed diverticulitis	0=no; 1=yes

Notes: Deficit points are summed for each individual, and divided by the total number of deficits, to produce a frailty index with a range from 0 to 1.

For peer review only - http://bmjopen.bmj.com/site/about/guidelines.xhtml

Supplementary Table 2. Regression models predicting frailty index

	Non-imputed data[1] (n=190,575)	Imputed data[1] (n=502,489)
Breastfed as a baby	-0.0042 (-0.0048,-0.0035)†	-0.0045 (-0.0051,-0.0038)†
Maternal smoking	0.0118 (0.0111,0.0125)†	0.0122 (0.0116,0.0128)†
Low birthweight	0.0108 (0.0097,0.0118)†	0.0114 (0.0105,0.0122)†
High birthweight	0.0030 (0.0021,0.0039)†	0.0036 (0.0028,0.0044)†
Perinatal diseases	0.0117 (0.0049,0.0185)*	0.0107 (0.0046,0.0167)*
Birth month (cos)	-0.0006 (-0.0011,-0.000)*	-0.0006 (-0.0010,-0.0002)*
Born in the UK	0.0024 (0.0011,0.0037)†	0.0018 (0.0006,0.0030)*
Education	-0.0140 (-0.0147,-0.0134)†	-0.0144 (-0.0150,-0.0139)†
Age (years)	0.0015 (0.0015,0.0015)†	0.0015 (0.0015,0.0016)†
Male	-0.0084 (-0.0090,-0.0078)†	-0.0084 (-0.0090,-0.0079)†
Caucasian ethnicity	-0.0069 (-0.0088,-0.0050)†	-0.0068 (-0.0085,-0.0051)†
Smoking	0.0244 (0.0234,0.0254)†	0.0248 (0.0239,0.0257)†
Alcohol drinking	-0.0260 (-0.0272,-0.0248)†	-0.0268 (-0.0279,-0.0258)†
Physical activity	-0.0065 (-0.0068,-0.0062)†	-0.0069 (-0.0072,-0.0067)†
Intercept	0.0976 (0.0947,0.1005)†	0.0998 (0.0972,0.1024)†

Note: [1] presented are coefficients (95% confidence intervals); *Significant at 0.05; † Significant at 0.0001. Non-imputed analysis was based on 214,104 respondents with complete information on all variables. The maternal smoking variable includes 13.86% missing data, the breastfed as a baby variable includes 23.64% missing data, the birthweight variable includes 44.88% missing data, the education variable includes 2.02% missing data, and the moderate or vigorous physical activity variable includes 2.43% missing data. The imputed analysis included all the respondents (n=502,489).

For peer review only

Reporting checklist for cross sectional study.

Based on the STROBE cross sectional guidelines.

Instructions to authors

Complete this checklist by entering the page numbers from your manuscript where readers will find each of the items listed below.

Your article may not currently address all the items on the checklist. Please modify your text to include the missing information. If you are certain that an item does not apply, please write "n/a" and provide a short explanation.

Upload your completed checklist as an extra file when you submit to a journal.

In your methods section, say that you used the STROBE cross sectionalreporting guidelines, and cite them as:

von Elm E, Altman DG, Egger M, Pocock SJ, Gotzsche PC, Vandenbroucke JP. The Strengthening the Reporting of Observational Studies in Epidemiology (STROBE) Statement: guidelines for reporting observational studies.

For peer review only

		Reporting Item	Page Number
**Title and abstract**			
Title	#1a	Indicate the study's design with a commonly used term in the title or the abstract	1
Abstract	#1b	Provide in the abstract an informative and balanced summary	3

		of what was done and what was found	
**Introduction**			
Background / rationale	#2	Explain the scientific background and rationale for the investigation being reported	4
Objectives	#3	State specific objectives, including any prespecified hypotheses	6
**Methods**			
Study design	#4	Present key elements of study design early in the paper	7
Setting	#5	Describe the setting, locations, and relevant dates, including periods of recruitment, exposure, follow-up, and data collection	7
Eligibility criteria	#6a	Give the eligibility criteria, and the sources and methods of selection of participants.	7
	#7	Clearly define all outcomes, exposures, predictors, potential confounders, and effect modifiers. Give diagnostic criteria, if applicable	8-9
Data sources / measurement	#8	For each variable of interest give sources of data and details of methods of assessment (measurement). Describe comparability of assessment methods if there is more than one group. Give information separately for for exposed and unexposed groups if applicable.	9
Bias	#9	Describe any efforts to address potential sources of bias	10
Study size	#10	Explain how the study size was arrived at	8

For peer review only

Quantitative variables	#11	Explain how quantitative variables were handled in the analyses. If applicable, describe which groupings were chosen, and why	8-9
Statistical methods	#12a	Describe all statistical methods, including those used to control for confounding	9-10
Statistical methods	#12b	Describe any methods used to examine subgroups and interactions	10
Statistical methods	#12c	Explain how missing data were addressed	10
Statistical methods	#12d	If applicable, describe analytical methods taking account of sampling strategy	8
Statistical methods	#12e	Describe any sensitivity analyses	10
**Results**			
Participants	#13a	Report numbers of individuals at each stage of study—eg numbers potentially eligible, examined for eligibility, confirmed eligible, included in the study, completing follow-up, and analysed. Give information separately for for exposed and unexposed groups if applicable.	7
Participants	#13b	Give reasons for non-participation at each stage	n/a
Participants	#13c	Consider use of a flow diagram	n/a
Descriptive data	#14a	Give characteristics of study participants (eg demographic,	11

For peer review only

		clinical, social) and information on exposures and potential confounders. Give information separately for exposed and unexposed groups if applicable.	
Descriptive data	#14b	Indicate number of participants with missing data for each variable of interest	11
Outcome data	#15	Report numbers of outcome events or summary measures. Give information separately for exposed and unexposed groups if applicable.	11
Main results	#16a	Give unadjusted estimates and, if applicable, confounder-adjusted estimates and their precision (eg, 95% confidence interval). Make clear which confounders were adjusted for and why they were included	11-12
Main results	#16b	Report category boundaries when continuous variables were categorized	11-12
Main results	#16c	If relevant, consider translating estimates of relative risk into absolute risk for a meaningful time period	12
Other analyses	#17	Report other analyses done—e.g., analyses of subgroups and interactions, and sensitivity analyses	12-13
**Discussion**			
Key results	#18	Summarise key results with reference to study objectives	13
Limitations	#19	Discuss limitations of the study, taking into account sources of potential bias or imprecision. Discuss both direction and magnitude of any potential bias.	14-15

For peer review only

Interpretation	#20	Give a cautious overall interpretation considering objectives, limitations, multiplicity of analyses, results from similar studies, and other relevant evidence.	14-15
Generalisability	#21	Discuss the generalisability (external validity) of the study results	14

Other Information

Funding	#22	Give the source of funding and the role of the funders for the present study and, if applicable, for the original study on which the present article is based	16

The STROBE checklist is distributed under the terms of the Creative Commons Attribution License CC-BY. This checklist was completed on 17. September 2021 using https://www.goodreports.org/, a tool made by the EQUATOR Network in collaboration with Penelope.ai

For peer review only

BMJ Open

Education mediating the associations between early life factors and frailty: a cross-sectional study of the UK Biobank

Journal:	*BMJ Open*
Manuscript ID	bmjopen-2021-057511.R2
Article Type:	Original research
Date Submitted by the Author:	24-Jan-2023

Complete List of Authors:	Maharani, Asri; The University of Manchester, Division of Population Health, Health Services Research & Primary Care; Manchester Metropolitan University, Department of Nursing<br>Didikoglu, Altug; The University of Manchester, Division of Neuroscience & Experimental Psychology<br>O'Neill, Terence; University of Manchester, Centre for Epidemiology Versus Arthritis, Division of Musculoskeletal & Dermatological Sciences; Manchester University NHS Foundation Trust, UK & NIHR Biomedical Research Centre<br>Pendleton, Neil; The University of Manchester, Division of Neuroscience & Experimental Psychology<br>Canal, Maria; The University of Manchester, Division of Neuroscience & Experimental Psychology<br>Payton, Antony; The University of Manchester, Division of Informatics, Imaging & Data Sciences

**Primary Subject Heading**:	Geriatric medicine
Secondary Subject Heading:	Epidemiology, Public health
Keywords:	EPIDEMIOLOGY, GERIATRIC MEDICINE, SOCIAL MEDICINE

SCHOLARONE™
Manuscripts

I, the Submitting Author has the right to grant and does grant on behalf of all authors of the Work (as defined in the below author licence), an exclusive licence and/or a non-exclusive licence for contributions from authors who are: i) UK Crown employees; ii) where BMJ has agreed a CC-BY licence shall apply, and/or iii) in accordance with the terms applicable for US Federal Government officers or employees acting as part of their official duties; on a worldwide, perpetual, irrevocable, royalty-free basis to BMJ Publishing Group Ltd ("BMJ") its licensees and where the relevant Journal is co-owned by BMJ to the co-owners of the Journal, to publish the Work in this journal and any other BMJ products and to exploit all rights, as set out in our licence.

The Submitting Author accepts and understands that any supply made under these terms is made by BMJ to the Submitting Author unless you are acting as an employee on behalf of your employer or a postgraduate student of an affiliated institution which is paying any applicable article publishing charge ("APC") for Open Access articles. Where the Submitting Author wishes to make the Work available on an Open Access basis (and intends to pay the relevant APC), the terms of reuse of such Open Access shall be governed by a Creative Commons licence – details of these licences and which Creative Commons licence will apply to this Work are set out in our licence referred to above.

Other than as permitted in any relevant BMJ Author's Self Archiving Policies, I confirm this Work has not been accepted for publication elsewhere, is not being considered for publication elsewhere and does not duplicate material already published. I confirm all authors consent to publication of this Work and authorise the granting of this licence.

For peer review only

Title Page

Title:

Education mediating the associations between early life factors and frailty: a cross-sectional study of the UK Biobank

Running Title:

Early-life factors and frailty

Keywords:

UK Biobank, Early-life factors, frailty

Authors and author affiliations:

Asri Maharani, MD, PhD[1*], Altug Didikoglu, PhD[2*], Terence W O'Neill, MD, PhD[3], Neil Pendleton, MD, PhD[2], Maria Mercè Canal, PhD[2], Antony Payton, PhD[4]

1) Division of Nursing, Midwifery, and Social Work, The University of Manchester, UK

2) Division of Neuroscience & Experimental Psychology, The University of Manchester, UK

3) Centre for Epidemiology Versus Arthritis, Division of Musculoskeletal & Dermatological Sciences, The University of Manchester, UK & NIHR Biomedical Research Centre, Manchester University NHS Foundation Trust, Manchester.

4) Division of Informatics, Imaging & Data Sciences, The University of Manchester, UK

* These authors contributed equally to this work

Corresponding author's full address and corresponding author's current email:

Name: Altug Didikoglu

Address: Division of Neuroscience & Experimental Psychology, The University of Manchester, AV Hill Building, Oxford Road, M13 9PL, Manchester, UK

Email: altug.didikoglu@manchester.ac.uk

Acknowledgements

For peer review only

Altug Didikoglu was supported by a grant from the Republic of Turkey Ministry of National Education. This research has been conducted using the UK Biobank Resource under Application Number 41877.

Conflicts of interest

All authors declare no conflicts of interest.

Main text word counts: 3492

Number of data elements: 4

For peer review only

For peer review only - http://bmjopen.bmj.com/site/about/guidelines.xhtml

Abstract

Objectives Exposures in utero and during infancy may impact the development of diseases later in life. They may be linked with development of frailty though the mechanism is unclear. This study aims to determine the associations between early life risk factors and development of frailty amongst middle-aged and older adults as well as potential pathways via education, for any observed association.

Design A cross-sectional study.

Settings This study used data from UK Biobank, a large population-based cohort.

Participants 502,489 individuals aged 37-73 years were included in the analysis.

Primary and secondary outcome measures Early life factors in this study included being breastfed as a baby, maternal smoking, birth weight, the presence of perinatal diseases, birth month, and birth place (in or outside the UK). We developed a frailty index comprising 49 deficits. We used generalised structural equation modelling to examine the associations between early life factors and development of frailty and whether any observed association was mediated via educational attainment.

Results A history of breastfeeding and normal birth weight were associated with a lower frailty index while maternal smoking, the occurrence of perinatal diseases and birth month with a longer day length were associated with a higher frailty index. Educational level mediated the relationship between these early life factors and frailty index.

Conclusions This study highlights that biological and social risk occurring at different stages of life are related to the variations in frailty index in later life and suggests opportunities for prevention across the life course.

Keywords early life factors, frailty, generalised structural equation model, UK Biobank

For peer review only - http://bmjopen.bmj.com/site/about/guidelines.xhtml

Article Summary

Strengths and limitations of this study

- Using a large cohort of British adults in middle and older age, this study was sufficiently powered to identify associations between early life factors and frailty index.
- The questionnaire on early life factors was based on self-report and is therefore subject to recall error.
- The information on the health conditions of the parents is limited, and the data on the breast feeding duration is not available.
- As the cohort is not nationally representative, the findings cannot be generalised to the general population.

For peer review only

For peer review only - http://bmjopen.bmj.com/site/about/guidelines.xhtml

Introduction

As the world's population ages, a major goal is the attainment of increased life expectancy accompanied by fewer years spent in poor health and with disability and dependency. The worldwide population of older people (65 years and above) is predicted to double from 0.7 billion (9%) in 2019 to 1.5 billion (16%) in 2050 [1]. In addition, there is evidence that the number of disability adjusted life years (DALY) among those aged 60 years and older is increasing (from 434 million in 1990 to 574 million in 2010) [2], which will increase demand for health and care services. As physical disability is an adverse outcome of frailty [3], more research in geriatrics and gerontology has focused on defining and recognising frailty among older people with the aim of determining preventive and interventional measures [4].

Frailty can be defined as a state of increased vulnerability resulting from an age-related decline in physiologic and cognitive reserves and function following stressor events [5]. The frailty index approach, developed by Rockwood et al. [6], measures frailty level as the number of deficits presents over the number of deficits considered, including symptoms, diagnoses, disabilities, and functional impairments. Frailty has become more common with the ageing of the population. A systematic review including 240 studies from 62 countries showed that 24% of people aged 50 years and older are frail as calculated using the frailty index approach [7]. Frailty has been found to be associated with adverse health outcomes including loss of mobility, disability, falls, hospitalisation, need for long-term care, and death [8-10]. Understanding the factors that are associated with frailty is thus important for developing interventions to prevent frailty and for providing directions for future public health policies.

A growing body of literature acknowledges that the first two decades of human life are critical in determining adult life trajectories. Among the early life factors, body size at birth [11-12], cigarette smoke exposure in utero [13], infants exclusively breastfed [14], birth month [15], and the presence of perinatal diseases [16] have been found to be associated with adult chronic

For peer review only - http://bmjopen.bmj.com/site/about/guidelines.xhtml

diseases. However, the study linking those factors and frailty is limited. In addition, the evidence on the link between early life factors and occurrence of frailty have been mixed [17,18]. The present study thus aims to determine the associations between early life factors, including a history of being breastfed, maternal smoking, birth weight, the presence of perinatal diseases, birth in or outside of the UK or outside the UK and birth month, and frailty in UK adults.

Furthermore, this study contributes to the literature investigating the determinants of health in later life by exploring the pathways of early life factors that have a lasting impact on health in middle and old age. The pathway hypothesis posits that early life conditions are important not only because they are directly associated with late life but also because they shape later life experiences [19,20], including restricted educational attainment and life chances. The most frequently hypothesised pathway between circumstances in early stages of life and adult health is adult socioeconomic status. Pakpahan et al. showed that socioeconomic factors in adulthood, including education, mediate the link between childhood health and socioeconomic conditions and self-rated health among older Europeans [19]. Because interventions that target common pathways have the potential to reduce frailty, the identification of the pathways of early life factors leading to frailty later in life has substantial public health relevance for the translation of life course epidemiology into practice. The present study considers whether any observed association between early life factors and frailty could be attributed to differences in education attainment (Figure 1).

Methods

Source and Sample

Data were drawn from the UK Biobank, a prospective cohort study of the genetic, environmental and lifestyle causes of diseases among adults in the UK [21]. The study involved the collection of extensive questionnaire data and biological samples from, and the

For peer review only - http://bmjopen.bmj.com/site/about/guidelines.xhtml

For peer review only

performance of, physical examinations of more than 500,000 respondents enrolled at 22 assessment sites in England, Scotland, and Wales between 2006 and 2010. Subjects who took part provided written informed consent for data collection, analysis and linkage; they also completed a touchscreen questionnaire, a nurse-led interview, and had their physical measurements taken. The UK Biobank invited adults who were registered with a general practitioner and who lived within reasonable traveling distance of the assessment centre. The current study includes 502,489 individuals aged 37-73 years who had study-specific available data and were not withdrawn from the study. This study was conducted as part of UK Biobank Project Number 41877 and is covered by the generic ethics approval for UK Biobank studies from the NHS National Research Ethics Service (16/NW/0274).

Measures

Early life factors

Information by questionnaire was obtained on: maternal smoking in the pre- and post-natal period, history of being breastfed as a baby, birth month, birthweight, the presence of perinatal diseases, and place of birth. We defined maternal smoking based on the question 'Did your mother smoke regularly around the time when you were born?' (Data-Field 1787). Respondents were categorised as having been breastfed as babies if they answered 'yes' to the question: 'Were you breastfed when you were a baby?' (Data-Field 1677). We retrieved information on birth month from the birth date (Data-Field 52) and treated it as the cosine of the values, representing the rhythmic seasonal length of day and night. We considered this might represent daylight time better than treating it as a categorical variable. This is an approach which we have used in a previous study [22]. Birth months of participants born in the UK and other countries in the southern hemisphere were converted to their antiphase. Information on birthweight was gathered by means of self-reported birthweight in kilograms (Data-Field 20022). We categorised the birth weight into low birth weight (<2,500 g), normal

For peer review only - http://bmjopen.bmj.com/site/about/guidelines.xhtml

birth weight (2,500 – 4,000 g), and high birth weight (>4,000 g). The presence of perinatal diseases ('ICD10 Chapter XVI: Certain conditions originating in the perinatal period') was coded as one based on self-reported medical history (Category 2416). We categorised the place birth of the respondents as born in the UK or outside the UK (Data-Field 1647). Answers of 'Do not know' or 'Prefer not to answer' were accepted as missing for all questions.

Education

The education variable represents the highest educational level completed by the respondents. Qualifications were categorised as high school or less (reference) and college or university degree (Data-Field 6138).

Frailty index

Following William et al. [23], we derived the frailty index using 49 functional, psychological, and social deficits within the range of data variables in the UK Biobank (see Supplementary Table 1). We coded the binary variables as 0 or 1, and for ordinal and continuous variables, coding was based on distribution. The total number of deficits was summed and divided by total possible deficits to create a frailty index between 0 and 1, where higher scores indicated greater frailty.

Covariates

We included demographic and health behaviour as covariates. Demographic information included age (in years; Data-Field 21003), gender (with male as the reference; Data-Field 31), and ethnicity (other than Caucasian as the reference or Caucasian; Data-Field 21000). Health behaviours included physical activity, alcohol intake and smoking status. Physical activity was measured as the number of days per week respondents engaged in at least 10 minutes of moderate or vigorous physical activity (Data-Field 884, Data-Field 904). Respondents were classified as non-current smokers (reference) or current smokers (Data-Field 20116). Alcohol

For peer review only - http://bmjopen.bmj.com/site/about/guidelines.xhtml

intake status was classified as non-current (reference) or current alcohol drinking (Data-Field 20117).

Statistical Analyses

Descriptive statistics were used to summarise subject characteristics including means and standard deviation for continuous variables and frequencies and percentages for categorical variables. We looked at the associations between frailty index and both early life factors and other covariates using unpaired t-tests (dichotomous variables), ANOVA (categorical variables), and Pearson's correlation (continuous variables).

We first performed a multivariate regression model including early life factors, education, and covariates (age, gender, ethnicity, smoking, alcohol drinking, physical activity). We further handled missing data using multivariate imputation by chained equations (MICE) [24] (using Stata's mi program) [25]. Twenty imputations were used.

The structural equation model (SEM) has been widely used to investigate complex relationships between variables in epidemiological studies [26]. SEM can be used to resolve the endogeneity problem between variables and to explore direct, indirect, and total effects between exogenous and endogenous variables. It can jointly test a variety of hypotheses that involve different types of complicated cause-effect relationships. However, all responses are assumed to be continuous, even when a variable is binary or categorical. In our analysis we include binary (education). To address this, we used a generalised structural equation model (GSEM) to identify the link between early life factors and frailty index and the mediating effect of education and income on that relationship. A GSEM combines generalised linear model (GLM) estimation and SEM modelling estimation; it can accommodate binary, ordinal, counted and categorical data [27]. Using maximum likelihood estimators, GLM estimators are based on a density function, allowing the direct use of all types of data [28]. The analyses were performed using MPlus version 8. We examined education as mediators of the

For peer review only - http://bmjopen.bmj.com/site/about/guidelines.xhtml

relationship in the GSEM model, which were controlled for age, gender, ethnicity and health behaviours (Model fit information: Chi-square=5049.35, p value=0.00; RMSEA=0.06, CFI=0.82; WRMR=13.01).

Patient and public involvement

Patients and/or the public were not involved in this study.

Results

Subjects

The study sample consisted of 502,489 respondents with an average age of 56.53 years (standard deviation [SD]=8.10 years) (Table 1). Just under half (45%) of the respondents were male, and most were Caucasian (94.59%). Around one-third of the respondents had graduated from college or university. The proportion of respondents whose mothers smoked regularly around the time of their birth was 29%. More than 72% of respondents were breastfed as babies, and 0.18% had perinatal diseases. 10% of respondents had low birth weight, while 13% of them had high birth weight. 91% of the respondents were born in the UK. Just over two-thirds of subjects reported engaging in at least 10 minutes of moderate or vigorous physical activity at least three days per week; 92% consumed alcohol and 11% were current smokers.

Table 1. Subject characteristics (n=502,489)

Variable	Percentage or mean (SD)*	Mean (SD) of frailty index**	Bivariate association with frailty index***
Frailty index, mean (SD)	0.14(0.08)		
Early-life factors			
Maternal smoking around birth, %			p<0.0001
No	70.75%	0.133(0.073)	
Yes	29.25%	0.146(0.078)	
Breastfed as a baby, %			p<0.0001
No	27.65%	0.137(0.076)	

For peer review only - http://bmjopen.bmj.com/site/about/guidelines.xhtml

Yes	72.35%	0.134(0.074)	
Birthweight, %			p<0.0001
Low birth weight	10.26%	0.149(0.080)	
Normal birth weight	76.34%	0.131(0.073)	
High birth weight	13.40%	0.136(0.076)	
Birth month, %			p=0.0002
January	8.44%	0.138(0.076)	
February	7.96%	0.137(0.075)	
March	8.98%	0.138(0.075)	
April	8.59%	0.139(0.076)	
May	8.98%	0.138(0.076)	
June	8.45%	0.139(0.076)	
July	8.48%	0.139(0.076)	
August	8.24%	0.138(0.076)	
September	8.14%	0.138(0.075)	
October	8.06%	0.137(0.076)	
November	7.63%	0.137(0.075)	
December	8.03%	0.138(0.076)	
Perinatal diseases, %			p<0.0001
No	99.82%	0.138(0.075)	
Yes	0.18%	0.149(0.084)	
Born in the UK, %			p=0.0381
No	8.96%	0.137(0.076)	
Yes	91.04%	0.138(0.075)	
Sociodemographics			
Age (years), mean (SD)	56.53(8.10)		R=0.16, p<0.0001
Gender, %			p<0.0001
Female	54.40%	0.141(0.075)	
Male	45.60%	0.134(0.076)	
Ethnicity, %			p<0.0001
Other	5.41%	0.141(0.078)	
Caucasian	94.59%	0.138(0.075)	
Education, %			p<0.0001
Less than college	67.27%	0.145(0.077)	
College or university degree	32.73%	0.122 (0.069)	
Health behaviours			
Moderate or vigorous physical activity, %			p<0.0001
None	10.75%	0.160(0.085)	
1 day	7.11%	0.134(0.072)	
2 days	13.40%	0.133(0.072)	
3 days or more	68.75%	0.135(0.073)	
Current alcohol consumption, %			p<0.0001
No	8.08%	0.166(0.088)	
Yes	91.92%	0.135(0.074)	
Current smoking, %			p<0.0001
No	89.39%	0.135(0.074)	
Yes	10.61%	0.159(0.084)	

For peer review only - http://bmjopen.bmj.com/site/about/guidelines.xhtml

Note: * Presented are means (standard deviation) for continuous variables and percentages for categorical variables. The maternal smoking variable includes 13.86% missing data, the breastfed as a baby variable includes 23.64% missing data, the birthweight variable includes 44.88% missing data, the education variable includes 2.02% missing data, and the moderate or vigorous physical activity variable includes 2.43% missing data. ** Presented are the means (standard deviation) of the frailty index per group. *** Bivariate analyses are unpaired t-tests for binary variables, ANOVA for ordinal variables, and Pearson's correlation for continuous variables.

Early life factors, covariates and frailty index

In bivariate analyses, compared to those whose mothers did not smoke around birth, maternal pre- and post-natal smoking was associated with a significantly higher frailty index (0.146 vs 0.133) as was the presence of perinatal diseases (0.149 vs 0.138) and being born in the UK (0.138 vs 0.137). A history of breast feeding was associated with a lower frailty index (0.134 vs 0.137). Low (0.149 vs 0.131) and high (0.136 vs 0.138) birthweight were associated with higher frailty scores compared to normal birthweight. Shorter daylight hours at birth (r=-0.01) were associated with lower frailty indices. As expected, the frailty index was higher among women than among men and in those with lower educational attainment. The frailty index was also higher in smokers, non-drinkers and those who engaged in less physical activity.

In regression analyses the effects of early life factors and covariates on the frailty index appeared similar in terms of both magnitude and direction when using both non-imputed and imputed data (see Supplementary Table 2). In these multivariate regression analyses adjusting for age, gender and health behaviours, birth month with longer hours of daylight, having a low and high birthweight, maternal smoking, being breastfed as baby, perinatal diseases and born in the UK had positive and significant associations with frailty index.

Mediation analysis

In the GSEM model, education mediated the association between early life factors and frailty index among middle-aged and older adults, supporting the pathway hypothesis. Table 2 presents the total, direct and indirect effects for each of the early life factors on the frailty

For peer review only - http://bmjopen.bmj.com/site/about/guidelines.xhtml

index. Maternal smoking (direct effect: coef.=0.068, z=33.40; indirect effect: coef.=0.011, z=25.54) and low birthweight (direct effect: coef.=0.041, z=20.93; indirect effect: coef.=0.003, z=9.18) and high birthweight (direct effect: coef.=0.013, z=6.34; indirect effect: coef.=0.001, z=4.09) directly and indirectly affected the frailty index compared to normal birthweight. The direct and indirect effects of being breastfed as a baby on having a lower frailty index were -0.022 (z=-10.36) and -0.009 (z=-22.91). Perinatal diseases had significant direct effect on higher frailty index (coef.=0.007, z=3.83), but it had no indirect effect on the frailty index (coef.=0.000, z=0.27). Being born in the UK, differently, had a significant indirect effect on higher frailty index (coef.=0.016, z=31.24), but it had no direct effect on the frailty index (coef.=0.002, z=0.74). Birth months with a short daylength were directly (coef.=-0.006, z=-2.91) and indirectly (coef.=-0.001, p-value=-2.35) associated with lower frailty scores.

Table 2. Total, direct, and indirect effects of early life factors on frailty index

	**Total effects**	**Direct effects**	**Indirect effects**
Breastfed as a baby	-0.031 (0.002)†	-0.022 (0.002)†	-0.009 (0.000)†
Maternal smoking around birth	0.079 (0.002)†	0.068 (0.002)†	0.011 (0.000)†
Low birth weight	0.045 (0.002)†	0.041 (0.002)†	0.003 (0.000)†
High birth weight	0.015 (0.002)†	0.013 (0.002)†	0.001 (0.000)†
Birth month (cos)	-0.007 (0.002)*	-0.006 (0.002)*	-0.001 (0.000)*
Perinatal diseases	0.007 (0.002)†	0.007 (0.002)†	0.000 (0.000)
Born in the UK	0.018 (0.002)†	0.002 (0.002)	0.016 (0.001)†

Education mediated the links between early life factors and frailty index (Figure 2). Participants born in the UK had a lower probability of completing higher education (coef.=-0.130, z=-44.65). Having been breastfed as a baby (coef.=0.076, z=26.87) was associated with higher educational attainment, while maternal smoking was associated with lower educational attainment (coef.=-0.087, z=-31.41). Both low (coef.=-0.027, z=-9.39) and high birthweight (coef.=-0.011, z=-4.11) was related to lower education attainment compared to normal

For peer review only - http://bmjopen.bmj.com/site/about/guidelines.xhtml

birthweight. Birth months with short daylight was related to higher education with the lowest effect size (coef.=0.006, z=2.35). Higher education was also associated with a lower frailty index (coef.=-0.123, z=-44.30). Amongst covariates with greater effect sizes, older age (coef.=0.178, z=83.25), lower activity levels (coef.=-0.088, z=-45.61) and smoking (coef.=0.106, z=56.36) were associated with a higher frailty index. Drinking alcohol is related to lower frailty index (coef.=-0.093; z=-51.63).

Discussions

Using data from UK Biobank we found that a history of breastfeeding was associated with a lower frailty index, while maternal smoking, having low or high birth weight, perinatal diseases and birth month with longer day length were associated with a higher frailty index. This study provides the first evidence that educational attainment level mediates the association between early life factors and frailty index.

Early life factors have previously been linked with higher frailty and chronic disease risk later in life [29,30]. Our findings highlight the importance of early life factors in determining frailty in middle age and older individuals. Maternal smoking was directly associated with higher frailty compared to those who were not exposed to maternal smoking. Evidence has suggested that cigarette smoke exposure in utero is linked to the development of chronic diseases later in life, including type 2 diabetes, obesity, certain cancers and respiratory disorders [13]. We also showed that this association was mediated by educational attainment. This is in line with a previous study which reported lower academic achievements of adolescents whose mothers smoked during pregnancy [31]. Maternal smoking during pregnancy was also found to be correlated with the children's cognitive function [32].

There is some evidence of a link between early life factors and occurrence of frailty. In a recent study in Finland, greater weight, length and BMI at birth were associated with a lower risk of frailty later in life [17]. In our study, having low or high birth weight were associated

For peer review only - http://bmjopen.bmj.com/site/about/guidelines.xhtml

with higher frailty index compared to having a normal birthweight, both directly and indirectly through education. Bleker and colleagues found that prenatal undernutrition was not associated with frailty but was associated with poorer health in old age, including slower gait speed and lower physical functioning which are components of the frailty phenotype, and the findings remained significant after inclusion of an extensive set of control variables including adult socioeconomic status [18]. Low birth weight is associated with increased risk of age-related diseases in prior review, and insulin-like growth factor (IGF-1) is the key driver of this process [33]. High birth weight may be the results of maternal obesity [34] and a study in Finland found that being born large for gestational age at term was associated with thicker carotid intima medial as the marker of subclinical atherosclerosis [35]. We also found that individuals who reported that they were breastfed have a lower frailty score. Infants exclusively breastfed have been found to have a lower risk of obesity, type 2 diabetes and high blood pressure in adulthood [14].

Birth month is associated with lower frailty index scores with a limited effect size in our study. In a large study in the US with 1,749,400 individuals showed that spring summer-born individuals have a relatively higher cardiovascular disease risk than autumn-winter born individuals and these seasons coincide with lower life expectancy [36]. This study showed that not only cardiovascular diseases, but several chronic diseases were associated with season of birth, having a different seasonal pattern. The underlying mechanisms may differ for each of these associations, such as sensitization to allergens or vitamin D deficiency [36]. Another possible mechanism is that differential light exposure during perinatal period influences development of the biological clock, in turn influencing later-life circadian rhythms and the sleep system, which are essential for health [37]. In European countries, it was shown that spring/summer born participants compared to autumn had higher frailty scores but this effect seemed independent of education [38]. However, we found and indirect effect of season of

For peer review only - http://bmjopen.bmj.com/site/about/guidelines.xhtml

birth through education. The indirect relationship of season of birth and frailty may be due to social factors such as the UK September date cut-off for starting education, which is in line with our findings showing an association between winter-born individuals and higher educational attainment [39,40].

Our results further suggest that having a perinatal disease was associated directly with higher frailty index scores. This finding is in keeping with that new-borns' perinatal complications are related to accelerated ageing at midlife [16]. Being born in the UK affected the frailty index indirectly through education, but not directly. Respondents who were not born in the UK were likely to have higher education attainment, which may enable better maintenance of health during older ages. However, we should note that our sample in this analysis may not be representative of the general population, and that participants were categorised as being born outside the UK without taking into account the country of origin and their socioeconomic background. In our analysis, we observed that education levels mediate the link between the other early life factors and the frailty index. Early life factors have a significant relationship with educational attainment, and higher education attainment is linked to a lower frailty index. This result is broadly in keeping with a prior study in Sweden which found that the associations between childhood conditions and various old age health indicators (musculoskeletal disorders, cardiovascular disease, self-rated health and impaired mobility) are mediated by education [41]. Prior research on the biological and psychological pathways linked childhood health and socioeconomic conditions to self-reported health status among older adults in 15 European countries [19]. Prior studies have shown that the life-course trajectories of socioeconomic attainment could be altered by physical and social conditions [42], and both childhood and adult conditions may impact health decades later [43]. Our findings have potential implications for policies aiming at preventing frailty among older adults. Subsequent circumstances mediate the impact of early life factors on frailty later in

For peer review only - http://bmjopen.bmj.com/site/about/guidelines.xhtml

life, and our study suggests that interventions such as improving education in midlife may mitigate early life disadvantages.

Our findings are based on a large and well characterised cohort. There are, however, a number of limitations to be consider in interpreting the results. First, information concerning early life factors in this study was based on self-report and is therefore subject to recall error. The likely effect of such error would be to underestimate the relationship between these factors and the frailty index. Second, we have limited access to the health conditions of the parents. A broad range of conditions which are comprised in the frailty index bear a hereditary risk, thus taking into account the health conditions of the parents is important in assessing the independent associations with frailty. Future studies may include the health conditions of the parents as the covariates. Third, the information on breast feeding duration is unavailable. Breast feeding for weeks rather than months may confer different outcomes. A dose response relationship thus cannot be assessed. Finally, these data were based on a sample of predominantly Caucasian men and women and should be extrapolated beyond this group with caution [44].

In conclusion, this study indicates an association between early life factors and frailty later in life. Early life conditions are important as the start of a mediated, incremental process during the life course. A comprehensive understanding of the determinants of frailty among middle-aged and older adults requires attention to exposures throughout the entire life course, with a special focus on the in utero and infancy stages and the chains of associated socioeconomic conditions that that connect over the life course. Applying a life course perspective to health in adulthood and old age should have implications for public health interventions, social policy, and further research. Early life is not the only period for any potential successful intervention; as our findings show, early life disadvantages may be offset by education and material wealth. Interventions throughout the life course, and especially during early life, could substantially reduce the health burden later in life.

For peer review only - http://bmjopen.bmj.com/site/about/guidelines.xhtml

Contributorship statement

A.D and A.M performed the data analysis. A.D and A.M drafted the manuscript. N.P, T.W.O, M.M.C., A.P. and were involved in planning and supervised the work. All authors discussed the results and commented on the manuscript.

Competing interest

All authors declare no conflicts of interest.

Funding

Altug Didikoglu was supported by a grant from the Republic of Turkey Ministry of National Education.

Data sharing statement

Data are available in a public, open access repository. Other researchers can apply for UK Biobank data to answer specific research questions.

Ethics Approval

This study was conducted as part of UK Biobank Project Number 41877 and is covered by the generic ethics approval for UK Biobank studies from the NHS National Research Ethics Service (16/NW/0274).

For peer review only

For peer review only - http://bmjopen.bmj.com/site/about/guidelines.xhtml

References

1. United Nations. World Population Prospects 2019: Highlights. New York: Department of Economic and Social Affairs; 2019. Contract No.: (ST/ESA/SER.A/423).
2. Prince MJ, Wu F, Guo Y, et al. The burden of disease in older people and implications for health policy and practice. *Lancet*. 2015;385:549-562.
3. Sternberg SA, Schwartz AW, Karunananthan S, Bergman H, Clarfield MA. The identification of frailty: a systematic literature review. *J Am Geriatr Soc*. 2011;59:2129-2138.
4. Daniels R, van Rossum E, de Witte L, Kempen GI, van den Heuvel W. Interventions to prevent disability in frail community-dwelling elderly: a systematic review. *BMC Health Serv Res*. 2008;8:278.
5. Hogan DB. Models, definitions, and criteria for frailty. *Conn's Handbook of Models for Human Aging*. 2018:35-44.
6. Rockwood K, Mitnitski A. Frailty defined by deficit accumulation and geriatric medicine defined by frailty. *Clin Geriatr Med*. 2011;27:17-26.
7. O'Caoimh R, Sezgin D, O'Donovan MR, et al. Prevalence of frailty in 62 countries across the world: a systematic review and meta-analysis of population-level studies. *Age and Ageing*. 2021;50:96-104.
8. Hanlon P, Nicholl BI, Jani BD, Lee D, McQueenie R, Mair FS. Frailty and prefrailty in middle-aged and older adults and its association with multimorbidity and mortality: a prospective analysis of 493 737 UK Biobank participants. *Lancet Public Health*. 2018;3:e323-e332.
9. Cegri F, Orfila F, Abellana RM, Pastor-Valero M. The Impact of Frailty on Admission to Home Care Services And Nursing Homes: Eight-Year Follow-Up of A Community-Dwelling, Older Adult, Spanish Cohort. *BMC Geriatrics*. 2020;20:1-13.
10. Ensrud KE, Ewing SK, Taylor BC, et al. Frailty and risk of falls, fracture, and mortality in older women: the study of osteoporotic fractures. *J Gerontol A Biol Sci Med Sci*. 2007;62:744-51.
11. Barker DJ. Fetal origins of coronary heart disease. *BMJ*. 1995;311:171-4.
12. Eriksson JG. Developmental origins of health and disease–from a small body size at birth to epigenetics. *Ann Med*. 2016;48:456-467.

For peer review only - http://bmjopen.bmj.com/site/about/guidelines.xhtml

13. Doherty S, Grabowski J, Hoffman C, Ng S, Zelikoff J. Early life insult from cigarette smoke may be predictive of chronic diseases later in life. *Biomarkers*. 2009;14(sup1):97-101.
14. Horta BL, Loret de Mola C, Victora CG. Long-term consequences of breastfeeding on cholesterol, obesity, systolic blood pressure and type 2 diabetes: a systematic review and meta-analysis. *Acta Paediatr*. 2015;104:30-7.
15. Zhang Y, Devore EE, Strohmaier S, Grodstein F, Schernhammer ES. Birth month, birth season, and overall and cardiovascular disease mortality in US women: prospective cohort study. *BMJ*. 2019;367.
16. Shalev I, Caspi A, Ambler A, et al. Perinatal complications and aging indicators by midlife. *Pediatrics*. 2014;134:e1315-e23.
17. Haapanen M, Perälä M, Salonen M, et al. Early life determinants of frailty in old age: the Helsinki Birth Cohort Study. *Age and Ageing*. 2018;47:569-75.
18. Bleker LS, de Rooij SR, Painter RC, van der Velde N, Roseboom TJ. Prenatal undernutrition and physical function and frailty at the age of 68 years: the Dutch Famine Birth Cohort Study. *J Gerontol A Biol Sci Med Sci*. 2016;71:1306-14.
19. Pakpahan E, Hoffmann R, Kröger H. The long arm of childhood circumstances on health in old age: Evidence from SHARELIFE. *Advances in Life Course Research*. 2017;31:1-10.
20. Power C, Hertzman C. Social and biological pathways linking early life and adult disease. *Br Med Bull*. 1997;53(1):210-21.
21. Sudlow C, Gallacher J, Allen N, Beral V, Burton P, Danesh J, et al. UK biobank: an open access resource for identifying the causes of a wide range of complex diseases of middle and old age. *PLoS Medicine*. 2015;12(3):e1001779.
22. Didikoglu A, Maharani A, Pendleton N, Canal MM, Payton A. Early life factors and COVID-19 infection in England: A prospective analysis of UK Biobank participants. *Early Human Development*. 2021;155:105326.
23. Williams DM, Jylhävä J, Pedersen NL, Hägg S. A frailty index for UK Biobank participants. *J Gerontol A Biol Sci Med Sci*. 2019;74(4):582-7.
24. Azur MJ, Stuart EA, Frangakis C, Leaf PJ. Multiple imputation by chained equations: what is it and how does it work? *International Journal of Methods in Psychiatric Research*. 2011;20(1):40-9.
25. StataCorp. Stata Multiple-Imputation Reference Manual Release 17. College Station, TX: StataCorp LLC; 2021.

For peer review only - http://bmjopen.bmj.com/site/about/guidelines.xhtml

26. Amorim LDAF, Fiaccone RL, Santos CAS, Santos TNd, de Moraes LTL, Oliveira NF, et al. Structural Equation Modeling in Epidemiology. *Cad Saúde Pública*. 2010;26(12):2251-62.
27. Zhang L, Zhang J. Perception of small tourism enterprises in Lao PDR regarding social sustainability under the influence of social network. *Tourism Management*. 2018;69:109-20.
28. McCullagh P, Nelder JA. *Generalized Linear Models*: Routledge; 2019.
29. Michels KB. Early life predictors of chronic disease. *J Womens Health*. 2003;12(2):157-61.
30. Li Y, Xue Q-L, Odden MC, Chen X, Wu C. Linking early life risk factors to frailty in old age: evidence from the China Health and Retirement Longitudinal Study. *Age and Ageing*. 2020;49(2):208-17.
31. Kristjansson AL, Thomas S, Lilly CL, Thorisdottir IE, Allegrante JP, Sigfusdottir ID. Maternal smoking during pregnancy and academic achievement of offspring over time: a registry data-based cohort study. *Preventive Medicine*. 2018;113:74-9.
32. Julvez J, Ribas-Fitó N, Torrent M, Forns M, Garcia-Esteban R, Sunyer J. Maternal smoking habits and cognitive development of children at age 4 years in a population-based birth cohort. *International Journal of Epidemiology*. 2007;36(4):825-32.
33. Vaiserman AM. Birth weight predicts aging trajectory: A hypothesis. *Mechanisms of Ageing and Development*. 2018;173:61-70.
34. Hanson M, Cooper C, Aihie Sayer A, Eendebak R, Clough G, Beard J. Developmental aspects of a life course approach to healthy ageing. *The Journal of Physiology*. 2016;594(8):2147-60.
35. Skilton MR, Viikari JS, Juonala M, Laitinen T, Lehtimäki T, Taittonen L, et al. Fetal growth and preterm birth influence cardiovascular risk factors and arterial health in young adults: the Cardiovascular Risk in Young Finns Study. *Arteriosclerosis, Thrombosis, and Vascular Biology*. 2011;31(12):2975-81.
36. Boland MR, Shahn Z, Madigan D, Hripcsak G, Tatonetti NP. Birth month affects lifetime disease risk: a phenome-wide method. *Journal of the American Medical Informatics Association*. 2015;22(5):1042-53.
37. Didikoglu A, Nair N, Robinson AC, Roncaroli F, Pendleton N, Payton A, et al. The effect of season of birth on brain epigenome-wide DNA methylation of older adults. *Journal of Developmental Origins of Health and Disease*. 2022;13(3):367-77.

For peer review only - http://bmjopen.bmj.com/site/about/guidelines.xhtml

38. Abeliansky AL, Strulik H. Season of birth, health and aging. *Economics & Human Biology*. 2020;36:100812.

39. Verachtert P, De Fraine B, Onghena P, Ghesquière P. Season of birth and school success in the early years of primary education. *Oxford Review of Education*. 2010;36(3):285-306.

40. Department of Education. Month of Birth and Education. Schools Analysis and Research Division. 2010. Contract No.: Research Report DFE-RR017.

41. Berndt H, Fors S. Childhood living conditions, education and health among the oldest old in Sweden. *Ageing & Society*. 2016;36(3):631-48.

42. Haas SA, Glymour MM, Berkman LF. Childhood health and labor market inequality over the life course. *Journal of Health and Social Behavior*. 2011;52(3):298-313.

43. Montez JK, Hayward MD. *Early life conditions and later life mortality*. International Handbook of Adult Mortality: Springer; 2011. p. 187-206.

44. Fry A, Littlejohns TJ, Sudlow C, Doherty N, Adamska L, Sprosen T, et al. Comparison of sociodemographic and health-related characteristics of UK Biobank participants with those of the general population. *American Journal of Epidemiology*. 2017;186(9):1026-34.

For peer review only

List of the figures and tables

Figure 1 The pathways of early life factors and impact on frailty among adults

Figure 2 Generalised structural equation models to identify the association between early life factors and frailty index, and education as mediators of the relationship between early life factors and frailty index. Note: *Significant at 0.05; † Significant at 0.0001

Table 1 Subject characteristics

Table 2 Total, direct, and indirect effects of early life factors on frailty index

For peer review only

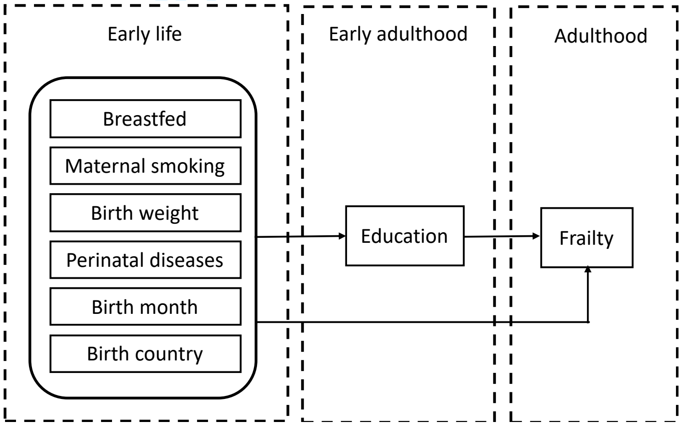

For peer review only

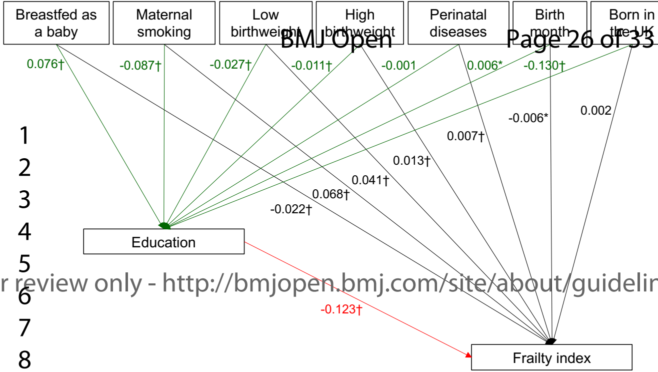

er review only - http://bmjopen.bmj.com/site/about/guideline

Supplementary Material: Pathways linking early life factors and frailty among middle-aged and older adults in England: Findings from UK Biobank

Supplementary Table 1. Variables included in the UK Biobank frailty indices

Item	Variable	Definition	Coding
	Sensory		
1	Glaucoma	Self-report of physician-diagnosed glaucoma	0=no; 1=yes
2	Cataracts	Self-report of physician-diagnosed glaucoma	0=no; 1=yes
3	Hearing difficulty	Self-report experiencing hearing difficulty	0=no; 1=yes/completely deaf
	Cranial		
4	Migraine	Self-report of physician-diagnosed migraine	0=no; 1=yes
5	Dental problems	Self-report of physician-diagnosed dental problems, i.e., ulcers, painful gums, bleeding gums, loose teeth, toothache, dentures	0=none; 1=any
	Mental well-being		
6	Self-rated health	Self-rated health in 4 Likert scale	0=excellent; 0.25=good; 0.5=fair; 1=poor
7	Fatigue	Self-report of frequency of tiredness / lethargy in last two weeks	0=not at all; 0.25=several days; 0.5=more than half; 1=nearly every day
8	Sleep	Self-report experiencing of sleeplessness/ insomnia	0=never/rarely; 0.5=sometimes; 1=usually
9	Depressed feelings	Self-report of frequency having depressed feeling in last two weeks	0=not at all; 0.5=several days; 0.75=more than half; 1=nearly every day
10	Self-described nervous personality	Self-report of having nervous personality	0=no; 1=yes
11	Severe anxiety/ panic attacks	Self-report of physician-diagnosed severe anxiety/panic attacks	0=no; 1=yes
12	Common to feel loneliness	Self-report of feeling lonely commonly	0=no; 1=yes
13	Sense of misery (ever/never)	Self-report of ever having sense of misery	0=no; 1=yes
	Infirmity		
14	Infirmity	Self-report of having long-standing illness or disability	0=no; 1=yes
15	Falls in last year	Self-report of experiencing falls last year	0=no falls; 0.5=one fall; 1=more than one fall
16	Fractures/broken bones in last five years	Self-report of experiencing fractures/broken bones in last five years	0=no; 1=yes
	Cardiometabolic		
17	Diabetes	Self-report of physician-diagnosed diabetes	0=no; 1=yes
18	Myocardial infarction	Self-report of physician-diagnosed myocardial infarction	0=no; 1=yes
19	Angina	Self-report of physician-diagnosed angina	0=no; 1=yes
20	Stroke	Self-report of physician-diagnosed stroke	0=no; 1=yes
21	High blood pressure	Self-report of physician-diagnosed high blood pressure	0=no; 1=yes
22	Hypothyroidism	Self-report of physician-diagnosed hypothyroidism	0=no; 1=yes
23	Deep-vein thrombosis	Self-report of physician-diagnosed deep-vein thrombosis	0=no; 1=yes
24	High cholesterol	Self-report of physician-diagnosed high cholesterol	0=no; 1=yes

For peer review only

For peer review only - http://bmjopen.bmj.com/site/about/guidelines.xhtml

	Respiratory		
25	Breathing	Self-report of having wheeze in last year	0=no; 1=yes
26	Pneumonia	Self-report of physician-diagnosed pneumonia	0=no; 1=yes
27	Chronic bronchitis/emphysema	Self-report of physician-diagnosed chronic bronchitis/emphysema	0=no; 1=yes
28	Asthma	Self-report of physician-diagnosed asthma	0=no; 1=yes
	Musculoskeletal		
29	Rheumatoid arthritis	Self-report of physician-diagnosed rheumatoid arthritis	0=no; 1=yes
30	Osteoarthritis	Self-report of physician-diagnosed osteoarthritis	0=no; 1=yes
31	Gout	Self-report of physician-diagnosed gout	0=no; 1=yes
32	Osteoporosis	Self-report of physician-diagnosed osteoporosis	0=no; 1=yes
	Immunological		
33	Hay fever, allergic rhinitis or eczema	Self-report of physician-diagnosed hay fever, allergic rhinitis or eczema	0=no; 1=yes
34	Psoriasis	Self-report of physician-diagnosed psoriasis	0=no; 1=yes
	Cancer		
35	Any cancer diagnosis	Self-report of physician-diagnosed any cancer	0=no; 1=yes
36	Multiple cancers diagnosed (number reported)	Self-report of physician-diagnosed multiple cancer	0=no cancer or single cancer; 1=multiple cancer
	Pain		
37	Chest pain	Self-report of ever experiencing chest pain	0=no; 1=yes
38	Head and/or neck pain	Self-report of ever experiencing head and/or neck pain	0=no; 1=yes
39	Back pain	Self-report of ever experiencing back pain	0=no; 1=yes
40	Stomach/abdominal pain	Self-report of ever experiencing stomach/abdominal pain	0=no; 1=yes
41	Hip pain	Self-report of ever experiencing hip pain	0=no; 1=yes
42	Knee pain	Self-report of ever experiencing knee pain	0=no; 1=yes
43	Whole-body pain	Self-report of ever experiencing whole-body pain	0=no; 1=yes
44	Facial pain	Self-report of ever experiencing facial pain	0=no; 1=yes
45	Sciatica	Self-report of physician-diagnosed sciatica	0=no; 1=yes
	Gastrointestinal		
46	Gastric reflux	Self-report of physician-diagnosed gastric reflux	0=no; 1=yes
47	Hiatus hernia	Self-report of physician-diagnosed hiatus hernia	0=no; 1=yes
48	Gall stones	Self-report of physician-diagnosed gall stones	0=no; 1=yes
49	Diverticulitis	Self-report of physician-diagnosed diverticulitis	0=no; 1=yes

Notes: Deficit points are summed for each individual, and divided by the total number of deficits, to produce a frailty index with a range from 0 to 1.

For peer review only

For peer review only - http://bmjopen.bmj.com/site/about/guidelines.xhtml

Supplementary Table 2. Regression models predicting frailty index

	**Non-imputed data[1] (n=190,575)**	**Imputed data[1] (n=502,489)**
Breastfed as a baby	-0.0042 (-0.0048,-0.0035)†	-0.0045 (-0.0051,-0.0038)†
Maternal smoking	0.0118 (0.0111,0.0125)†	0.0122 (0.0116,0.0128)†
Low birthweight	0.0108 (0.0097,0.0118)†	0.0114 (0.0105,0.0122)†
High birthweight	0.0030 (0.0021,0.0039)†	0.0036 (0.0028,0.0044)†
Perinatal diseases	0.0117 (0.0049,0.0185)*	0.0107 (0.0046,0.0167)*
Birth month (cos)	-0.0006 (-0.0011,-0.000)*	-0.0006 (-0.0010,-0.0002)*
Born in the UK	0.0024 (0.0011,0.0037)†	0.0018 (0.0006,0.0030)*
Education	-0.0140 (-0.0147,-0.0134)†	-0.0144 (-0.0150,-0.0139)†
Age (years)	0.0015 (0.0015,0.0015)†	0.0015 (0.0015,0.0016)†
Male	-0.0084 (-0.0090,-0.0078)†	-0.0084 (-0.0090,-0.0079)†
Caucasian ethnicity	-0.0069 (-0.0088,-0.0050)†	-0.0068 (-0.0085,-0.0051)†
Smoking	0.0244 (0.0234,0.0254)†	0.0248 (0.0239,0.0257)†
Alcohol drinking	-0.0260 (-0.0272,-0.0248)†	-0.0268 (-0.0279,-0.0258)†
Physical activity	-0.0065 (-0.0068,-0.0062)†	-0.0069 (-0.0072,-0.0067)†
Intercept	0.0976 (0.0947,0.1005)†	0.0998 (0.0972,0.1024)†

Note: [1] presented are coefficients (95% confidence intervals); *Significant at 0.05; † Significant at 0.0001. Non-imputed analysis was based on 214,104 respondents with complete information on all variables. The maternal smoking variable includes 13.86% missing data, the breastfed as a baby variable includes 23.64% missing data, the birthweight variable includes 44.88% missing data, the education variable includes 2.02% missing data, and the moderate or vigorous physical activity variable includes 2.43% missing data. The imputed analysis included all the respondents (n=502,489).

For peer review only

Reporting checklist for cross sectional study.

Based on the STROBE cross sectional guidelines.

Instructions to authors

Complete this checklist by entering the page numbers from your manuscript where readers will find each of the items listed below.

Your article may not currently address all the items on the checklist. Please modify your text to include the missing information. If you are certain that an item does not apply, please write "n/a" and provide a short explanation.

Upload your completed checklist as an extra file when you submit to a journal.

In your methods section, say that you used the STROBE cross sectionalreporting guidelines, and cite them as:

von Elm E, Altman DG, Egger M, Pocock SJ, Gotzsche PC, Vandenbroucke JP. The Strengthening the Reporting of Observational Studies in Epidemiology (STROBE) Statement: guidelines for reporting observational studies.

		Reporting Item	Page Number
**Title and abstract**			
Title	#1a	Indicate the study's design with a commonly used term in the title or the abstract	1
Abstract	#1b	Provide in the abstract an informative and balanced summary	3

For peer review only

		of what was done and what was found	
**Introduction**			
Background / rationale	#2	Explain the scientific background and rationale for the investigation being reported	4
Objectives	#3	State specific objectives, including any prespecified hypotheses	6
**Methods**			
Study design	#4	Present key elements of study design early in the paper	7
Setting	#5	Describe the setting, locations, and relevant dates, including periods of recruitment, exposure, follow-up, and data collection	7
Eligibility criteria	#6a	Give the eligibility criteria, and the sources and methods of selection of participants.	7
	#7	Clearly define all outcomes, exposures, predictors, potential confounders, and effect modifiers. Give diagnostic criteria, if applicable	8-9
Data sources / measurement	#8	For each variable of interest give sources of data and details of methods of assessment (measurement). Describe comparability of assessment methods if there is more than one group. Give information separately for for exposed and unexposed groups if applicable.	9
Bias	#9	Describe any efforts to address potential sources of bias	10
Study size	#10	Explain how the study size was arrived at	8

For peer review only

Quantitative variables	#11	Explain how quantitative variables were handled in the analyses. If applicable, describe which groupings were chosen, and why	8-9
Statistical methods	#12a	Describe all statistical methods, including those used to control for confounding	9-10
Statistical methods	#12b	Describe any methods used to examine subgroups and interactions	10
Statistical methods	#12c	Explain how missing data were addressed	10
Statistical methods	#12d	If applicable, describe analytical methods taking account of sampling strategy	8
Statistical methods	#12e	Describe any sensitivity analyses	10
**Results**			
Participants	#13a	Report numbers of individuals at each stage of study—eg numbers potentially eligible, examined for eligibility, confirmed eligible, included in the study, completing follow-up, and analysed. Give information separately for for exposed and unexposed groups if applicable.	7
Participants	#13b	Give reasons for non-participation at each stage	n/a
Participants	#13c	Consider use of a flow diagram	n/a
Descriptive data	#14a	Give characteristics of study participants (eg demographic,	11

For peer review only

		clinical, social) and information on exposures and potential confounders. Give information separately for exposed and unexposed groups if applicable.	
Descriptive data	#14b	Indicate number of participants with missing data for each variable of interest	11
Outcome data	#15	Report numbers of outcome events or summary measures. Give information separately for exposed and unexposed groups if applicable.	11
Main results	#16a	Give unadjusted estimates and, if applicable, confounder-adjusted estimates and their precision (eg, 95% confidence interval). Make clear which confounders were adjusted for and why they were included	11-12
Main results	#16b	Report category boundaries when continuous variables were categorized	11-12
Main results	#16c	If relevant, consider translating estimates of relative risk into absolute risk for a meaningful time period	12
Other analyses	#17	Report other analyses done—e.g., analyses of subgroups and interactions, and sensitivity analyses	12-13
**Discussion**			
Key results	#18	Summarise key results with reference to study objectives	13
Limitations	#19	Discuss limitations of the study, taking into account sources of potential bias or imprecision. Discuss both direction and magnitude of any potential bias.	14-15

For peer review only

Interpretation	#20	Give a cautious overall interpretation considering objectives, limitations, multiplicity of analyses, results from similar studies, and other relevant evidence.	14-15
Generalisability	#21	Discuss the generalisability (external validity) of the study results	14
**Other Information**			
Funding	#22	Give the source of funding and the role of the funders for the present study and, if applicable, for the original study on which the present article is based	16

The STROBE checklist is distributed under the terms of the Creative Commons Attribution License CC-BY. This checklist was completed on 17. September 2021 using https://www.goodreports.org/, a tool made by the EQUATOR Network in collaboration with Penelope.ai

For peer review only
